# Supplementary material for: Characterizing barren plateaus in quantum ansätze with the adjoint representation
Source: Nat Commun. 2024 Aug 22;15:7171. doi: 10.1038/s41467-024-49910-w (PMC11341719; doi:10.1038/s41467-024-49910-w)
Supplement: Supplementary file 1 — Supplementary Information [file 41467_2024_49910_MOESM1_ESM.pdf]

Supplementary Information: "Characterizing Barren Plateaus in Quantum Ansätze with the Adjoint Representation"

Enrico Fontana,[1,2] Dylan Herman,[1,*] Shouvanik Chakrabarti,[1] Niraj Kumar,[1]
Romina Yalovetzky,[1] Jamie Heredge,[1,3] Shree Hari Sureshbabu,[1] and Marco Pistoia[1]

[1] *Global Technology Applied Research, JPMorgan Chase*
[2] *Computer and Information Sciences, University of Strathclyde*
[3] *School of Physics, The University of Melbourne*

Contents

1. Introduction to Lie Groups and Representation Theory 1
   A. Lie Groups, Lie Algebras, Representations 2
   B. Useful Representations and Norms 3
   C. Casimir Operators 3
   D. Cartan Subalgebras, Weights, and Roots 4

2. Verifying Theory Reproduces Existing Results 5
   A. SU(d) 5
   B. SU($d/2$) $\times$ SU($d/2$) 6
   C. Spin–$\frac{d-1}{2}$ representation of SU(2) 6

3. Proofs of Technical Lemmas 7

4. Proof of Main Text Theorem 2.13 9

5. Projected Norm Lower Bound 12

6. Applicability of Theory beyond LASA 14
   A. Proof of Supplementary Theorem 5 16
   B. Variance of Non-ideal Complement 20

7. Details of Numerical Results 21

8. Mixing time to t-designs 21

   References 26

Supplementary Note 1 – Introduction to Lie Groups and Representation Theory

This section presents a short intro to the representation theory of Lie groups and provides sufficient background understanding the results of the paper and their proofs. The only prerequisite is knowledge of some concepts from algebra and topology. For a more detailed introduction the reader is directed to any one of the following fabulous texts [1–5].

---

* dylan.a.herman@jpmorgan.com

A. Lie Groups, Lie Algebras, Representations

A *Lie group*, G, is a topological group that is also a smooth manifold. The *Lie algebra*, $\mathfrak{g}$, associated with G is the tangent space at the group's identity element and forms a non-associative algebra with the Lie bracket operation, denoted $[\cdot,\cdot]$. Specifically, the Lie bracket obeys the Jacobi identity: $\forall h,k,j \in \mathfrak{g}$,

$$[h,[k,j]] + [k,[j,h]] + [j,[h,k]] = 0, \tag{1}$$

and is additionally bilinear and skew symmetric.

Our focus will be *compact Lie groups* (or subgroups of compact Lie groups), which are Lie groups whose topology is compact. In the compact setting, it is without loss of generality to restrict our attention to groups of matrices, called matrix Lie groups (see the Peter-Weyl theorem [1, Corollary 4.22]). This restriction also has the benefit of simplifying some of the more abstract notions mentioned above. First, one can now view the Lie bracket as the matrix commutator. Second, the matrix exponential maps elements of $\mathfrak{g}$ onto analytic curves in neighborhood of the identity, i.e. $\forall t \in \mathbb{R}, \gamma(t) = e^{t\cdot\mathbf{H}} \in G$ with $\mathbf{H} \in \mathfrak{g}$ being the tangent vector. Third, *compact Lie algebras*, those associated with a compact Lie group, can be assumed to only contain skew-Hermitian matrices. If the topology of G is both compact and connected, then the matrix exponential from $\mathfrak{g}$ to G is surjective, i.e. for any $g \in G, \exists h \in \mathfrak{g}$ s.t. $g = e^h$.

Since we will be only considering compact Lie groups, we can assume the existence of a finite Haar measure [6, 7], enabling integration over the whole group. This a uniform measure, μ, that is invariant under left and right translation by group elements, i.e. for some fixed $\mathfrak{g}$, $\mu(gh) = \mu(hg), \forall h \in G$. This enables one to integrate over the group and compute various stastical moments. Throughout the paper, all integration, e.g. $\int_G f(g)dg$, is with respect to the Haar measure

A *simple Lie algebra* is one that has dimension greater than one and no non-trivial ideals and is *semi-simple* if $\mathfrak{g}$ can be decomposed into a direct sum of simple Lie algebras. Note that (semi-)simple Lie groups have (semi-)simple Lie algebras.

Let V denote a real or complex, finite-dimensional inner product space, $\mathcal{U}(V)$ denote the group of isometries on V, and $\mathfrak{u}(V)$ denote the algebra of skew-Hermitian operators on V. Without loss of generality one could take of course this to be $\mathbb{C}^n$ for some n, up to isomorphism. A unitary *Lie group representation* of G is the following smooth homomorphism $\phi : G \to \mathcal{U}(V)$, and ϕ is called *faithful* if it is injective. The representation makes the space V into a G-module.

A *subrepresentation* of ϕ is an invariant subspace of V, and a representation is *irreducible* if it has no non-trivial subrepresentations. Compact Lie groups are guaranteed to have finite-dimensional unitary representations, which actually implies that any representation is *reducible*, i.e. can be decomposed as a direct sum of irreducible subrepresentations (also called irreducible components). More specifically, an the vector space on which G is acting can be equipped with an inner product that makes the action unitary. In addition, representations of simple Lie algebras are always faithful or trivial. We will frequently use the notation $\mathbf{U}_g$ to denote the element $\phi(g) \in \mathcal{U}(V)$ for some $g \in G$ when the representation ϕ and V are clear from the context.

A map f between representations ϕ and ψ is called *equivariant* if $\forall g \in G, f \circ \phi(g) = \psi(g) \circ f$, and if f is bijective, then the representations are *isomorphic as G-modules. Schur's lemma* states that any equivariant map between irreducible representations is either an isomorphism or the zero map. Furthermore, if the representation space is complex, then f must be a multiple of the identity and any two equivariant maps are scalar multiplies of one another.

The group representation induces a *Lie algebra representation* $d\phi : \mathfrak{g} \to \mathfrak{u}(V)$, which is the differential of the smooth map ϕ. Just as group representations respect the group operation (i.e. are homomorphims), Lie algebra representations respect addition and the Lie bracket (the commutator):

$$[d\phi(x), d\phi(y)] = d\phi([x,y]), \tag{2}$$

specifically it is an algebra homomorphism.

Given an orthornomal basis $\{v_j\}_j$ for V, for i,j, the associated *matrix coefficient* is defined as $\phi_{i,j}(g) = \langle \phi(g)v_i, v_j\rangle$. For example, the matrix coefficients of the adjoint representation are the structure constants with respect to the basis $\{v_j\}_j$ for $\mathfrak{g}$. *Schur orthogonality* states that for non-isomorphic irreducible representations ϕ and ψ:

$$\int_G \phi_{ij}(g)\overline{\psi_{kl}(g)}dg = 0, \tag{3}$$

and for the same representation with orthonormal basis $\{v_j\}_j$ for V_ϕ:

$$\int_G \phi_{ij}(g)\overline{\phi_{kl}(g)}dg = \frac{\delta_{ik}\delta_{jl}}{\dim V_\phi}. \tag{4}$$

B. Useful Representations and Norms

There are a few types of representations that we will refer to frequently. If a compact Lie group G contains $n \times n$ unitary matrices, its *standard representation* is the natural action on $V = \mathbb{C}^n$. The Lie algebra standard representation follows similarly but replacing unitary with skew-Hermitian. The *adjoint representation* consists of G acting on its Lie algebra by conjugation, i.e. ϕ is defined by $\phi(g)h = ghg^{-1}$, $\forall h \in \mathfrak{g}$, and $V_\phi = \mathfrak{g}$. The associated Lie algebra adjoint representation is defined by $d\phi(h)k = [h,k]$ $\forall k \in \mathfrak{g}$, and $V_\phi = \mathfrak{g}$. Lastly, the tensor power of a Lie group representation ϕ, denoted by $\phi \otimes \phi$, is defined as $\forall g \in G, (\phi \otimes \phi)(g) = \phi(g) \otimes \phi(g)$ and acts on $V^{\otimes 2}$. The tensor power of the associated Lie algebra representation, denoted $d\phi \otimes d\phi$, is defined as $\forall h \in \mathfrak{g}, (d\phi \otimes d\phi)(h) = d\phi(h) \otimes \mathbb{1} + \mathbb{1} \otimes d\phi(h)$ for identity operator $\mathbb{1}$.

Let $\mathrm{Tr}(\cdot)$ denote the standard trace for linear operators. Given a Lie algebra $\mathfrak{g}$, we can set an orthonormal basis for the Lie algebra $\{e_i\}$, where e_i are associated with the standard representation of $\mathfrak{g}$, using the *standard trace form*:

$$-\mathrm{Tr}(e_i e_j) = \delta_{ij}, \tag{5}$$

where the negative is to ensure that the form is positive definite. Each representation also has an associated trace form defined in a similar manner, i.e. by multiplying and taking traces of the matrices involved. Specifically, the *Killing form* is the trace form associated with the adjoint representation.

Note that the Lie algebra representation does not, necessarily, preserve these forms, meaning that $d\phi(e_i)$ is not normalized, w.r.t. the trace form for ϕ, in general. Still, it is a well-known result that for simple Lie algebras any trace form is a scalar multiple of the (nondegenerate) Killing form. Furthermore, for compact Lie algebras the trace forms are always real and definite. Thus every two trace forms are a *real* scalar multiple of each other. Thus, for any representation ϕ of a compact simple Lie group, we define a scaling constant I_ϕ that we call the *index of the representation* (w.r.t. the standard representation) such that:

$$-\mathrm{Tr}(d\phi(e_i) d\phi(e_j)) = I_\phi \delta_{ij}. \tag{6}$$

This is the same as (twice) the Dynkin index for irreducible representations [8]. We will omit the subscript in the trace when it is obvious which space it is taken in. The above discussion also implies that for compact simple Lie algebra, the trace forms induce valid inner produces and norms [1].

The first norm, which we call the *standard norm*, is one induced by the standard trace form. For any $a \in \mathfrak{g}$,

$$\|a\|_{\mathfrak{g}}^2 = -\mathrm{Tr}(a^2). \tag{7}$$

We call the norm induced by the Killing form, the *Killing norm* and denote it by $\|\cdot\|_{\mathrm{K}}$. In general, if we are working in a representation ϕ then we can define the Frobenius norm of $d\phi(a), a \in \mathfrak{g}$:

$$\|d\phi(a)\|_{\mathrm{F}}^2 = -\mathrm{Tr}(d\phi(a)^2). \tag{8}$$

All norms are related via the representation index

$$\|d\phi(a)\|_{\mathrm{F}} = I_\phi \|a\|_{\mathfrak{g}}^2 \tag{9}$$

$$\|d\phi(a)\|_{\mathrm{K}}^2 = \|a\|_{\mathrm{K}}^2 = I_{\mathrm{Ad}} \|a\|_{\mathfrak{g}}^2 = \frac{I_{\mathrm{Ad}}}{I_\phi} \|d\phi(a)\|_{\mathrm{F}}^2. \tag{10}$$

Note that the compact Lie algebras are completely classified, and I_{Ad} is $\Theta(\sqrt{d_{\mathfrak{g}}})$ for all non-exceptional classes (i.e. the dual Coxeter numbers) [9]. In addition, the equality $\|d\phi(a)\|_{\mathrm{K}}^2 = \|a\|_{\mathrm{K}}^2$ follows from the faithfulness of representations of simple Lie algebras.

C. Casimir Operators

As mentioned earlier, the commutator bracket is non-associative product. However, a Lie algebra can be embedded in a larger algebra is associative and equiped with an additional product. This algebra, denoted by $\mathcal{U}(\mathfrak{g})$ is called the *universal enveloping algebra* of $\mathfrak{g}$. The embedding map $i : \mathfrak{g} \to \mathcal{U}(\mathfrak{g})$ satisfies the additional important properly: $\forall h, k \in \mathfrak{g}$

$$[i(h), i(k)] = i(h)i(k) - i(k)i(h), \tag{11}$$

where juxtaposition represents the additional associative product of $\mathcal{U}(\mathfrak{g})$. Thus, $\mathcal{U}(\mathfrak{g})$ is an associative algebra where the Lie bracket acts as the commutator. We will from now on drop the map i and just use $\cdot$ denote this new associative product.

The algebra $\mathcal{U}(\mathfrak{g})$ is formed by quotienting the tensor algebra of $\mathfrak{g}$ so that above desired relation is satisfied. In addition, representations of $\mathcal{U}(\mathfrak{g})$ and $\mathfrak{g}$ are one-to-one with each other and respect the new product when extended.

There is an important element that lies in the center of $\mathcal{U}(\mathfrak{g})$ called the *quadratic Casimir operator* defined as:

$$c = \sum_j e_j \cdot e_j, \tag{12}$$

where $\{e_j\}$ forms a basis for $\mathfrak{g}$. With respect to algeba representations, the quadratic Casimir maps to:

$$\boldsymbol{C}_\phi = \sum_j d\phi(e_j) \cdot d\phi(e_j), \tag{13}$$

and under the tensor power of a representation it maps to

$$(d\phi \otimes d\phi)(c) = \sum_j (d\phi(e_j) \otimes \mathbb{1} + \mathbb{1} \otimes d\phi(e_j)) \cdot (d\phi(e_j) \otimes \mathbb{1} + \mathbb{1} \otimes d\phi(e_j)) \tag{14}$$

$$= 2\sum_j d\phi(e_j) \otimes d\phi(e_j) + (\boldsymbol{C}_\phi \otimes \mathbb{1} + \mathbb{1} \otimes \boldsymbol{C}_\phi). \tag{15}$$

The first term in the second equality is called the *split quadratic Casimir operator*:

$$\mathbf{K}_\phi = \sum_j d\phi(e_j) \otimes d\phi(e_j). \tag{16}$$

One may note that Supplementary Equation (13) and (16) have a similar appearance. In fact, as mentioned earlier, the associative product on $\mathcal{U}(\mathfrak{g})$ was obtained by quotienting the tensor algebra (i.e. $\otimes \mapsto \cdot$). Thus, in the tensor algebra, before quotienting, these two operators are formally identical. In fact, even Ref. [10] called what we refer to as the split Casimir the quadratic Casimir.

However, traditionally Supplementary Equation (13), as written, is what one refers to as the quadratic Casimir and from a representation theoretic point-of-view there are some technical differences between the two. Specifically, $\boldsymbol{C}_\phi$ is associated with the representation ϕ while $\mathbf{K}_\phi$ makes use of the notion of tensor power representation. As such (also shown in [11]), given irreducible ϕ, the corresponding split Casimir operator is proportional to the identity on the irreducible components of $\phi \otimes \phi$ but in general is not on ϕ. The version of the split Casimir considered in the main text contains an additional normalization by the representation index I_ϕ. Specifically, the $\mathbf{K}$ used in the main text is defined as $\mathbf{K} = I_\phi^{-1}\mathbf{K}_\phi$.

D. Cartan Subalgebras, Weights, and Roots

There is a particular convenient basis for $\mathfrak{g}$ consisting of elements that lie in its complexification, i.e. the algebra $\mathfrak{g}^{\mathbb{C}} := \mathfrak{g} + i\mathfrak{g}$. In addition, any representation of $d\phi$ can be uniquely linearly extended to a representation for the complexification, in the natural way, which we denote by $d\phi_{\mathbb{C}}$. This basis is called the Cartan–Weyl basis, which we denote as

$$\{H_i\}_{i=1}^r \cup \{E_{\vec{\alpha}}, E_{-\vec{\alpha}}\}_{\alpha\in\Delta^+}, \tag{17}$$

where $E_{\vec{\alpha}}$ and $E_{-\vec{\alpha}}$ can be chosen to be dual under the Killing form.

The set of elements $\{H_i\}_{i=1}^r$ are mutually commuting, which form a subalgebra called a *Cartan subalgebra*. Hence, if we consider a representation of $\mathfrak{g}$, $d\phi_{\mathbb{C}}$, then $\{d\phi_{\mathbb{C}}(H_i)\}_{i=1}^r$ are simultaneously diagonalizable linear operators. Thus to each simultaneous eigenvector $v \in V$ we can associate a real-valued linear functional $\vec{\omega}_v$, called a weight, that satisfies

$$d\phi_{\mathbb{C}}(H_i)v = \vec{\omega}_v(H_i)v \tag{18}$$

for $1 \le i \le r$. It is known that the weights can be viewed as r-dimensional vectors on a lattice. The set Δ^+ consists of a particular basis (as a lattice) for the weights called the positive simple weights. The other elements indexed by this set $E_{\vec{\alpha}}, E_{-\vec{\alpha}}$ satisfy the following fundamental property: if v is a weight vector with weight ω, then $\forall i$.

$$d\phi_{\mathbb{C}}(H_i)d\phi_{\mathbb{C}}(E_{\pm\vec{\alpha}})v = (\vec{\omega} \pm \vec{\alpha})(H_i)d\phi_{\mathbb{C}}(E_{\pm\vec{\alpha}})v. \tag{19}$$

Physicists may recognize these as raising and lowering operators. Then in this basis, the split Casimir is expressed as:

$$\mathbf{K} = I_\phi^{-1}\left(\sum_{i=1}^{r} d\phi_{\mathbb{C}}(H_i)\otimes d\phi_{\mathbb{C}}(H_i) + \sum_{\vec{\alpha}\in\Delta^+}\left[d\phi_{\mathbb{C}}(E_{\vec{\alpha}})\otimes d\phi_{\mathbb{C}}(E_{-\vec{\alpha}}) + d\phi_{\mathbb{C}}(E_{-\vec{\alpha}})\otimes d\phi_{\mathbb{C}}(E_{\vec{\alpha}})\right]\right). \tag{20}$$

Lastly, if V is an irreducible representation, it can be expressed as a direct sum of simultaneous, orthogonal eigenspaces of the $\{H_i\}_{i=1}^r$ called weight spaces. Specifically, if $V_{\vec{\omega}}$ is the weight space associated to weight $\vec{\omega}$ then $\forall v \in V_{\vec{\omega}}$ and $\forall i$:

$$d\phi_{\mathbb{C}}(H_i)v = \vec{\omega}(H_i)v, \tag{21}$$

generalizing Supplementary Equation (18).

As mentioned earlier the Killing form is positive definite on $\mathfrak{g}$ and thus can be used to induce a valid inner product on its dual space (which contains roots). More specifically, if T_{ij} is the Killing form, then for any two roots $\vec{\alpha}, \vec{\beta} \in \Delta^+$:

$$(\vec{\alpha}, \vec{\beta}) = [T^{-1}]_{ij}\alpha_i\beta_j. \tag{22}$$

We begin with a very brief review some of the relevant concepts from the representation theory of complex semisimple Lie algebras and direct the interested reader to standard references for more details [1–5].

Let $\{H_i\}_{i=1}^r$ denote a basis for $\mathfrak{h}$ that is orthonormal with respect to the Killing form (the Killing form is positive definite on $\mathfrak{h}$). Let $d\phi : \mathfrak{g} \to \mathfrak{u}(V)$ denote a finite-dimensional representation and $d\phi' : \mathfrak{g}^{\mathbb{C}} \to \mathfrak{gl}(V)$ the unique complex-linear extension. Since each $d\phi'(H_i)$ can be simultaneous diagonalized, to each simultaneous eigenvector $v \in V$ we associate a real-valued linear functional $\vec{\omega}_v$, called a weight, that satisfies $d\phi'(H_i)v = \vec{\omega}_v(H_i)v$. The vector v is called a weight vector, and the space of all vectors associated with the weight, is called a weight space. When $d\phi$ is the adjoint representation the functionals $\vec{\omega}_v$ are called roots.

There is a partial ordering on the weights, i.e. $\mu \geq \tau$ if $\mu - \tau$ has all positive coefficients in its expansion in terms of Δ_+. A consequence is that if $|\vec{\Lambda}\rangle$ is a highest weight vector (unique up to scaling), then $d\phi'(E_{\vec{\alpha}})|\vec{\Lambda}\rangle = 0$.

For a simple root $\vec{\alpha}_i$, its coroot is defined as

$$\alpha_i^\vee = \frac{2\vec{\alpha}^{(i)}}{(\vec{\alpha}^{(i)}, \vec{\alpha}^{(i)})}, \tag{23}$$

which is an element of the double dual space and hence identifiable with elements of $\mathfrak{h}$. Then the associated Gram matrix is [12]:

$$G_{ij} = (\alpha_i^\vee, \alpha_j^\vee) = \frac{2}{(\vec{\alpha}^{(i)}, \vec{\alpha}^{(i)})}\frac{2(\vec{\alpha}^{(i)}, \vec{\alpha}^{(j)})}{(\vec{\alpha}^{(j)}, \vec{\alpha}^{(j)})} = \frac{2}{(\vec{\alpha}^{(i)}, \vec{\alpha}^{(i)})}A_{ij}, \tag{24}$$

where A_{ij} is called the Cartan matrix. The dual basis to the set of coroots is called the Dynkin basis or set of fundamental weights. Thus if $\vec{\lambda}$ is a weight expressed in the Dynkin basis, then its norm can be expressed as

$$(\vec{\lambda}, \vec{\lambda}) = \sum_{ij} G_{ij}^{-1} n_i n_j, \tag{25}$$

where $\{n_i\}$ are the integer components of $\vec{\lambda}$ in the Dynkin basis.

Supplementary Note 2 – Verifying Theory Reproduces Existing Results

In this section, we apply the results from the main text to three cases where the expression for the gradient variance is already known in literature.

A. SU(d)

We should be able to replicate the result for ordinary barren plateaus, that is, the fully controllable case where we have the group SU(d) acting via the standard representation. In that case, the basis is the normalized multi-qubit basis giving $I_\phi = 1$. The Lie algebra $\mathfrak{su}(d)$ of dimension $d^2 - 1$. Since this is a complete orthonormal basis for traceless

Hermitian matrices, for a general Hermitian $\mathbf{A}$, $\|P_{\mathfrak{g}}\mathbf{A}\|_{\mathrm{F}}^2 = \mathrm{Tr}(\mathbf{A}_T^2)$. By assumption $\mathbf{O}$ is traceless and Hermitian and $\mathbf{H}$ is traceless and skew-Hermitian so $\|\mathbf{O}\|_{\mathfrak{g}}^2 = \mathrm{Tr}(\mathbf{O}^2)$ and $\|\mathbf{H}\|_{\mathfrak{g}}^2 = -\mathrm{Tr}(\mathbf{H}^2)$. Finally, $I_{\mathrm{Ad}} = 2d$ for $\mathfrak{su}(d)$. So

$$\mathrm{GradVar} = \frac{2d}{(d^2-1)^2}\mathrm{Tr}(\mathbf{O}^2)\mathrm{Tr}(\mathbf{H}^2)\mathrm{Tr}(\rho_T^2). \tag{26}$$

This agrees exactly with the subspace-controllable result, see Eq. 13 in [13].

B. $\mathbf{SU}(d/2) \times \mathbf{SU}(d/2)$

The quantum circuit realizing the standard representation of the semisimple $G = \mathrm{SU}(d/2) \times \mathrm{SU}(d/2) \subset \mathrm{SU}(d)$ is an example of a subspace-controllable system. The subalgebra is semisimple: $\mathfrak{g} = \mathfrak{g}_1 \oplus \mathfrak{g}_2$ where each subalgebra is isomorphic to $\mathfrak{su}(d/2)$. Since the system is embedded in $\mathrm{SU}(d)$, $I_{\phi_1} = I_{\phi_2} = 1$. The projector onto each subalgebra is thus equivalent to taking the partial trace of the (traceless) operator on the other subsystem, i.e. $\mathbf{H} = \mathbf{H}_1 \otimes \mathbb{1} + \mathbb{1} \otimes \mathbf{H}_2$, and $P_{\mathfrak{g}_i}\mathbf{H} = \mathbf{H}_i$. For a general Hermitian $\mathbf{A}$, $\|P_{\mathfrak{g}_i}A\|_{\mathrm{F}}^2 = \mathrm{Tr}([\mathrm{Tr}_{i^c}(A_T)]_T^2)$. Since we are in a LASA, we can decompose $\mathbf{O}$ and $\mathbf{H}$ into parts supported on each subalgebra. Thus finally

$$\mathrm{GradVar} = \sum_{i=1,2}\frac{2(d/2)}{((d/2)^2-1)^2}\mathrm{Tr}(\mathbf{H}_i^2)\mathrm{Tr}(\mathbf{O}_i^2)\mathrm{Tr}([\mathrm{Tr}_{i^c}(\rho_T)]_T^2). \tag{27}$$

For states supported on either subalgebra, this agrees with the aforementioned subspace-controllable result. If the traceless part of the state is a simple sum of parts on the subspaces, i.e. if it is unentangled between subspaces, then the variance is the sum of the variances over the subspaces. However, for a state that is maximally entangled the partial trace on each subspace will be proportional to the identity, and therefore $[\mathrm{Tr}_{i^c}(\rho_T)]_T = 0$, giving a zero total variance. This is consistent with the basic quantum information theory notion that local operators cannot distinguish between maximally-entangled subsystems.

C. Spin$-\frac{d-1}{2}$ representation of $\mathbf{SU}(2)$

Here we consider the case of the spin$-\frac{d-1}{2}$ irreducible representation, ϕ_d, of $\mathfrak{su}(2)$ in an n-qubit quantum system, $d = 2^n$. We have $d_{\mathfrak{g}} = 3$. Let us consider a normalized basis for the standard (spin 1/2) representation of the Lie algebra: $\{e_1, e_2, e_3\} = \{-\frac{i}{\sqrt{2}}\sigma_x, -\frac{i}{\sqrt{2}}\sigma_y, -\frac{i}{\sqrt{2}}\sigma_z\}$. Since these have commutation relations $[e_i, e_j] = \sqrt{2}\epsilon_{ijk}e_k$, we relate them to the canonical Hermitian spin$-\frac{d-1}{2}$ generators $\{S_x, S_y, S_z\}$ with commutation $[S_x, S_y] = iS_z$ via $\phi_d(e_1) = -i\sqrt{2}S_x$, etc. Recall that, as discussed in the main text, we can use the representation index to express the variance as

$$\mathrm{GradVar} = \frac{I_{\mathrm{Ad}}\|o\|_{\mathfrak{g}}^2\|h\|_{\mathfrak{g}}^2}{d_{\mathfrak{g}}^2}\sum_i \mathrm{Tr}^2(i\rho\mathbf{E}_i). \tag{28}$$

It is a well-known result that $I_{\mathrm{Ad}} = 2n$ for $\mathfrak{su}(n)$, and thus we have $I_{\mathrm{Ad}} = 4$ for this case. Now for the calculation of the norms. Choosing $\mathbf{H} = -iS_x$, we have $\|h\|_{\mathfrak{g}}^2 = \frac{1}{2}$. With $\mathbf{O} = S_x + S_y + S_z$, we get $\|o\|_{\mathfrak{g}}^2 = \frac{3}{2}$. Finally, $\rho = |m\rangle\langle m|$, giving

$$\sum_i \mathrm{Tr}^2(i\rho\mathbf{E}_i) = 2\mathrm{Tr}^2(|m\rangle\langle m|S_z) = 2(\langle m|S_z|m\rangle)^2 = 2m^2. \tag{29}$$

Overall using Eq. (28) we get the result

$$\mathrm{GradVar} = \frac{2m^2}{3}, \tag{30}$$

which agrees with the result for the same system found in [13].

Supplementary Note 3 – Proofs of Technical Lemmas

In this section, we provide the proofs of the technical lemmas that were necessary to derive Theorems 2.8 and 2.9 of the main text.

The first lemma shows that the first Haar moment is zero for simple groups, which allowed us to only focus on the second moment when computing the variance. This is a commonly observed regarding the first Haar moment of the gradient. However, we include a proof for the general case for completeness.

Supplementary Lemma 1 (Vanishing mean)**.** *Let G be a compact Lie group, and $\phi : G \to \mathcal{U}(V)$ a representation. Then for any $\mathbf{O}, \mathbf{A} \in \mathfrak{gl}(V)$:*

$$\mathbb{E}_{g^+,g^-\sim\mu^{\otimes 2}}[\partial\langle\mathbf{O}\rangle_{\mathbf{A}}] = 0. \tag{31}$$

Thus we have that for compact Lie groups the variance of the gradient equals its second moment, and so we only need focus on the latter.

Proof. In full, the first moment is

$$\mathbb{E}_{g^+,g^-\sim\mu^{\otimes 2}}[\partial\langle\mathbf{O}\rangle_{\mathbf{A}}] = \int_G \mathrm{Tr}\{\mathbf{U}_{g^-}\mathbf{A}\mathbf{U}^\dagger_{g^-}[\mathbf{H}, \mathbf{U}_{g^+}\mathbf{O}\mathbf{U}^\dagger_{g^+}]\}dg^+dg^- = \int_G \mathrm{Tr}\left\{\mathbf{U}_{g^-}\mathbf{A}\mathbf{U}^\dagger_{g^-}\left[\mathbf{H}, \int_G \mathbf{U}_{g^+}\mathbf{O}\mathbf{U}^\dagger_{g^+}dg^+\right]\right\}dg^-. \tag{32}$$

We can show that it is zero by proving that the commutator is zero for any $\mathbf{O}$. Since twirling projects onto the commutant of the group, $e^{\theta\mathbf{H}}$ commutes with the integral. This follows from the invariance of the Haar measure.

Let $\mathbf{U}_h = \phi(h)$ for $h \in G$. Then

$$\mathbf{U}_h\left(\int_G \mathbf{U}_g\mathbf{O}\mathbf{U}^\dagger_g dg\right) = \int_G \mathbf{U}_{(hg)}\mathbf{O}\mathbf{U}^\dagger_g dg = \int_G \mathbf{U}_{g'}\mathbf{O}\mathbf{U}^\dagger_{(h^{-1}g')}dg' = \left(\int_G \mathbf{U}_{g'}\mathbf{O}\mathbf{U}^\dagger_{g'}dg'\right)\mathbf{U}_h, \tag{33}$$

where we changed the variable of integration to $g' = hg$ (which leaves the integral unchanged due to Haar translation invariance) and used the fact that $\mathbf{U}^\dagger_g = \mathbf{U}_{g^{-1}}$.

Since differentiation commutes with linear operators, and the commutator is linear in each argument, this implies that

$$0 = \frac{d}{d\theta}\left[e^{\theta\mathbf{H}}, \int_G \mathbf{U}_{g^+}\mathbf{O}\mathbf{U}^\dagger_{g^+}dg^+\right]\Big|_{\theta=0} = \left[\frac{de^{\theta\mathbf{H}}}{d\theta}\Big|_{\theta=0}, \int_G \mathbf{U}_{g^+}\mathbf{O}\mathbf{U}^\dagger_{g^+}dg^+\right] = \left[\mathbf{H}, \int_G \mathbf{U}_{g^+}\mathbf{O}\mathbf{U}^\dagger_{g^+}dg^+\right]. \tag{34}$$

□

The next lemma was essential for computing the gradient variance for the simple group case (Theorem 2.8 in the main text):

Lemma 4.1. *Let G be a compact simple Lie group with Lie algebra $\mathfrak{g}$. Suppose V is a finite-dimensional inner product space, $\phi : G \to \mathcal{U}(V)$ is a unitary representation of G, and $\mathbf{U}_g = \phi(g)$. In addition, $a \in \mathfrak{g}$, $\mathbf{A} = d\phi(a)$. Then the following holds: Then*

$$\int_G (\mathbf{U}_g\mathbf{A}\mathbf{U}^\dagger_g)^{\otimes 2}\, dg = \frac{\|\mathbf{A}\|_F^2}{d_{\mathfrak{g}}}\mathbf{K}, \tag{35}$$

where $\mathbf{K}$ is the split Casimir.

Proof. Since differentiation commutes with linear maps, one can show that $\mathbf{U}_g\mathbf{A}\mathbf{U}^\dagger_g = d\phi(\mathrm{Ad}_g(a))$. Let $\{e_j\}$ be a basis for $\mathfrak{g}$, then $\{\mathbf{E}_j\}$, where each $\mathbf{E}_j = d\phi(e_j)$ is skew-Hermitian, is a basis for $d\phi(\mathfrak{g})$. Then, by linearity we have

$$\mathbf{U}_g\mathbf{A}\mathbf{U}^\dagger_g = \mathrm{Ad}_g(\mathbf{A}) = \sum_{ji} a_i[\mathrm{Ad}_g]_{ij}\mathbf{E}_j, \tag{36}$$

where

$$\mathbf{U}_g\mathbf{E}_i\mathbf{U}^\dagger_g = \sum_j [\mathrm{Ad}_g]_{ji}\mathbf{E}_j = d\phi\left(\sum_{ji}[\mathrm{Ad}_g]_{ji}e_j\right). \tag{37}$$

Explicitly, $\{[\mathrm{Ad}_g]_{ji}\}$ are the matrix coefficients for the adjoint representation of G, and are real since we are dealing with a real Lie algebra.

Since $a \in \mathfrak{g}$, we have that $a = \sum_i a_i e_i$. Then the LHS becomes

$$\int_G \mathrm{Ad}_g(\mathbf{A}) \otimes \mathrm{Ad}_g(\mathbf{A})\, dg = \sum_{ii'jj'} a_i a_{i'} \int_G [\mathrm{Ad}_g]_{ij} [\mathrm{Ad}_g]_{i'j'}\, dg\ \ (\mathbf{E}_j \otimes \mathbf{E}_{j'}). \tag{38}$$

Now let's use the fact that the adjoint representation is irreducible for simple Lie groups. This allows us to use Schur orthogonality [1, Corollary 4.10] to write

$$\int_G [\mathrm{Ad}_g]_{ij} [\mathrm{Ad}_g]_{i'j'}\, dg = \delta_{ii'} \delta_{jj'} \frac{1}{d_{\mathfrak{g}}}. \tag{39}$$

Note, the theorem requires conjugation of one of the terms, however all the coefficients in the adjoint representation are real so we can ignore this. In addition, since $\mathfrak{g}$ is compact, its complexification is simple and thus the complex extension of the adjoint representation is irreducible, allowing us to apply Schur orthogonality. This finally gives

$$\int_G (\mathbf{U}_g \mathbf{A} \mathbf{U}_g^\dagger)^{\otimes 2}\, dg = \frac{1}{d_{\mathfrak{g}}} \sum_i a_i^2 \ \sum_j \mathbf{E}_j \otimes \mathbf{E}_j. \tag{40}$$

By definition of $\mathbf{K}$, we have that $\sum_j \mathbf{E}_j \otimes \mathbf{E}_j = I_\phi \mathbf{K}$. □

The following is a generalization of Lemma 4.1 to outside the simple group setting and was needed for proving Theorem 2.9 of the main text.

Lemma 4.2. *Let G be a compact and connected Lie group with Lie algebra $\mathfrak{g}$. Suppose V is a finite-dimensional inner product space, $\phi : G \to \mathcal{U}(V)$ is a unitary representation of G, and $\mathbf{U}_g = \phi(g)$. In addition, $a \in \mathfrak{g}$, $\mathbf{A} = d\phi(a)$. Then the following holds:*

$$\int_G (\mathbf{U}_g \mathbf{A} \mathbf{U}_g^\dagger)^{\otimes 2} dg = \sum_\alpha \frac{\|\mathbf{A}_{\mathfrak{g}_\alpha}\|_F^2}{d_{\mathfrak{g}_\alpha}} \mathbf{K}_{\mathfrak{g}_\alpha} + \mathbf{A}_{\mathfrak{c}}^{\otimes 2}, \tag{41}$$

where $\mathbf{A}_\alpha$ is the image of the component of a in $\mathfrak{g}_\alpha$ under $d\phi$. Likewise, $\mathbf{K}_\alpha$ is the split Casimir in the subalgebra $\mathfrak{g}_\alpha$.

Proof. Since $\mathfrak{g}$ is reductive, the algebra's adjoint representation, ad breaks into a direct sum of irreducible representations, i.e. the simple ideals of $\mathfrak{g}$ and its center $\mathfrak{c}$. The simple ideals $\mathfrak{g}_\alpha$ must correspond to non-isomorphic simple $\mathfrak{g}$-modules, and since G is connected, they correspond to non-isomorphic simple G-modules. Furthermore, the center $\mathfrak{c}$ breaks up into a direct sum of trivial representations. Thus, the Schur orthogonality relations imply that cross terms are zero, and the integral breaks up:

$$\int_G (\mathbf{U}_g \mathbf{A} \mathbf{U}_g^\dagger)^{\otimes 2}\, dg = \left(\sum_\alpha \int_G \mathrm{Ad}_{\mathbf{U}_{g_\alpha}} (\mathbf{A}_{\mathfrak{g}_\alpha})^{\otimes 2}\, dg_\alpha \right) + \mathbf{A}_{\mathfrak{c}}^{\otimes 2} = \sum_\alpha \frac{\|\mathbf{A}_{\mathfrak{g}_\alpha}\|_{\mathrm{F}}^2}{d_{\mathfrak{g}_\alpha}} \mathbf{K}_{\mathfrak{g}_\alpha} + \mathbf{A}_{\mathfrak{c}}^{\otimes 2}, \tag{42}$$

where the last equality follows from applying Lemma 4.1 to the components of the direct sum. In addition $\mathbf{A}_{\mathfrak{g}_\alpha} \in d\phi(\mathfrak{g}_\alpha), \mathbf{A}_{\mathfrak{c}} \in d\phi(\mathfrak{c})$ and $\mathbf{A} = \sum_\alpha \mathbf{A}_{\mathfrak{g}_\alpha} + \mathbf{A}_{\mathfrak{c}}$. □

The above result implies that we expect contributions to the variance from the various subalgebras.

Lastly, for completeness, we also proof the following simple fact used in Section 2 E.

Supplementary Lemma 2. *For any $\mathbf{H}$ in Lie algebra $\mathfrak{g}$, we have*

$$\|\mathbf{H}\|_K^2 \leq 2 d_{\mathfrak{g}} \|\mathbf{H}\|_{\mathrm{F}}^2 \tag{43}$$

Proof. Let $\{\mathbf{E}_k\}_{k=1}^{d_{\mathfrak{g}}}$ be an orthonormal basis for $\mathfrak{g}$, then

$$\|\mathbf{H}\|_{\mathrm{K}}^2 = \sum_{j,k=1}^{d_{\mathfrak{g}}} \mathrm{Tr}([\mathbf{H}, \mathbf{E}_k] \mathbf{E}_j)^2 = \sum_{k=1}^{d_{\mathfrak{g}}} \|[\mathbf{H}, \mathbf{E}_k]\|_{\mathrm{F}}^2 \leq 2 d_{\mathfrak{g}} \|\mathbf{H}\|_{\mathrm{F}}^2. \tag{44}$$

□

Supplementary Note 4 – Proof of Main Text Theorem 2.13

As mentioned in the main text, the compound SU layers can also be viewed as the direct sum of the alternating representations of $\mathrm{SU}(n)$. The k-th alternating representation is $\phi_k : \mathrm{SU}(n) \to \mathcal{U}(\bigwedge^k \mathbb{C}^n)$ and is irreducible. The direct sum is then obviously $\phi : \mathrm{SU}(n) \to \mathcal{U}(\bigoplus_{k=1}^n \bigwedge^k \mathbb{C}^n)$. Let $\{e_k\}$ denote the standard basis for $\mathbb{C}^n$. The mapping between the qubit state space and $\bigwedge^k \mathbb{C}^n$ can be explicitly seen by mapping a computational basis state $|S\rangle \mapsto \bigwedge_{i\in[n]|S_i=1} e_i$. We will restrict our analysis to the $k = n/2$ subspace (if n is not even take $k = n/2+1$ or $k = n/2-1$), which has dimension exponential in n, i.e. $\binom{n}{n/2} = \Omega(2^{n/2})$. Since $\phi_{n/2}$ is faithful, the dimension of Lie algebra of $\phi_{n/2}(\mathrm{SU}(n))$ is the same as $\mathfrak{su}(n)$, i.e. n^2-1.

Since we will not be able to use the adjoint representation trick, we need to tackle computing the second-moment operator

$$\mathcal{T} : \mathbf{A} \mapsto \int_{\mathrm{SU}(n)} (\mathbf{U}_g \otimes \mathbf{U}_g)\mathbf{A}(\mathbf{U}_g^\dagger \otimes \mathbf{U}_g^\dagger) dg \tag{45}$$

directly using Schur–Weyl duality [3, Theorem 6.3]. Recall that $\mathcal{T}$ must respect the decomposition of the tensor product representation, i.e. $\wedge^{n/2}\mathbb{C}^n \otimes \wedge^{n/2}\mathbb{C}^n$, into irreducible components. For the current setting, the Pierri formula [3, Exercise 6.16] implies that the decomposition into irreducible components is

$$\wedge^{n/2}\mathbb{C}^n \otimes \wedge^{n/2}\mathbb{C}^n = \bigoplus_{a\in[n/2+1]} V_{\lambda_a}, \tag{46}$$

where λ_a denotes the partition of the integer n that has $n/2-a$ 2's and $2a$ 1's. Specifically, the λ_a index the Young diagrams on $2n$ boxes of shape λ_a. Furthermore, due to the form of the integral that appears when computing the variance of the gradient, we only need to consider even a. This is because if ρ is the initial state lying in the Hamming-weight $n/2$ subspace, we are considering the inner product between the integral and a symmetric tensor $\rho \otimes \rho \in \mathrm{Sym}^2(\wedge^{n/2}\mathbb{C}^n)$. Thus,

$$\mathrm{Sym}^2(\wedge^{n/2}\mathbb{C}^n) = \bigoplus_{a\in[n/2+1]\ \&\ a \text{ is even}} V_{\lambda_a}. \tag{47}$$

As consequence of Schur–Weyl duality for $\mathrm{SU}(n)$, there is a basis for $V_{\lambda_a} \subset \wedge^{n/2}\mathbb{C}^n \otimes \wedge^{n/2}\mathbb{C}^n$, known as the Gelfand–Cetlin basis [4, Cor 8.1.7], whose elements are in one-to-one correspondence with the *semistandard Young Tableau* (SSYT) of shape λ_a. While this is not an orthogonal basis and thus is not the basis that diagonalizes the HYOs, it will suffice for reasoning about which irreps the tensor $|S\rangle \otimes |S\rangle$ has support on when $|S\rangle$ is a computational basis state. This leads to the following lemma.

Supplementary Lemma 3. *If $|S\rangle$ is a computational basis state of Hamming weight k then $|S\rangle \otimes |S\rangle$ lies in an irreducible subrepresentation of $\wedge^k\mathbb{C}^n \otimes \wedge^k\mathbb{C}^n$, specifically V_{λ_0}.*

Proof. Given an SSYT of shape λ_a, an element of the Gelfand–Cetlin basis is formed by symmetrizing over the rows of the SSYT, where elements in the same column correspond to antisymmetrized indices. One can verify that if $|S\rangle$ is a computational basis state, $|S\rangle \otimes |S\rangle$ corresponds to an SSYT of shape $\lambda_0 = (2, \ldots, 2)$ and weight also $(2, \ldots, 2)$, i.e. k rows and two columns. This SSYT is already symmetric across the rows and so the row symmetrizer acts as identity. The conclusion is that the tensor product of a computational basis state with itself lies in V_{λ_0}. □

With regards to the integral, the result will be that the projections onto V_{λ_a} for $\lambda_a \neq \lambda_0$ will not contribute if our initial state is a computational basis state of Hamming weight k.

Another consequence of Schur–Weyl duality is that the dimension of V_{λ_0} is equal to the Schur polynomial for partition λ_0 evaluated at all 1's, i.e. $S_{\lambda_0}(1, \ldots, 1)$.

Supplementary Lemma 4. *Let λ_0 denote the partition $(2, \ldots, 2)$ of n, for some even integer n. Then,*

$$\dim V_{\lambda_0} = \binom{n}{n/2}^2 \frac{n+1}{(n/2+1)^2}. \tag{48}$$

Proof. Note that λ_0', the conjugate partition to λ_0, is given by $(n/2, n/2)$. To evaluate the Schur polynomials, we will use the second form of the Jacobi–Trudi identity [3, Equation A.6] which states that for any partition λ of n, the Schur polynomial S_λ is given by,

$$S_\lambda = \det(e_{\lambda_i'+j-i})_{i,j=1}^{l(\lambda')} \tag{49}$$

where λ' is the conjugate partition to λ, $l(\lambda')$ is its length, and e_k denotes the kth elementary symmetric polynomial on n variables. Recall that the elementary symmetric polynomial e_k is the sum of all monomials of total degree k, where no individual variable has degree greater than 1.

Specializing (49) to our case, we have that

$$S_{\lambda_0} = \det \begin{bmatrix} e_{n/2} & e_{n/2+1} \\ e_{n/2-1} & e_{n/2} \end{bmatrix} = e_{n/2}^2 - e_{n/2-1}e_{n/2+1}. \tag{50}$$

It remains to evaluate $e_{n/2}, e_{n/2-1}, e_{n/2+1}$ at the points of interest, which can be done in our case from simple counting arguments.

To evaluate the polynomials at $(1, 1, \ldots, 1)$, we note that each elementary polynomial e_k contains exactly $\binom{n}{k}$ monomials, each of which evaluates to 1 at $(1, 1, \ldots, 1)$. Thus $e_k(1, 1, \ldots, 1) = \binom{n}{k}$. Noticing additionally that $\binom{n}{n/2-1} = \binom{n}{n/2+1} = \frac{n/2}{n/2+1}\binom{n}{n/2}$, we have,

$$S_{\lambda_0}(1, 1, \ldots, 1) = \binom{n}{n/2}^2 - \binom{n}{n/2-1}\binom{n}{n/2+1} \tag{51}$$

$$= \binom{n}{n/2}^2 \left(1 - \frac{(n/2)^2}{(n/2+1)^2}\right) \tag{52}$$

$$= \binom{n}{n/2}^2 \frac{n+1}{(n/2+1)^2}. \tag{53}$$

□

We now have tools to prove Theorem 2.13 from the main text, which we restate below.

Supplementary Theorem 1 (Main text Theorem 2.13)**.** *For the quantum compound ansatz if the initial state is a computational basis state with Hamming-weight $\frac{n}{2}$ and the observable is a rank-one projector onto another computational basis state in this space, then*

$$\text{GradVar} \in \mathcal{O}\left(\binom{n}{n/2}^{-1}\right). \tag{54}$$

Proof. Recall that $\text{GradVar} = \mathbb{E}_{g^+,g^-\sim\mu^{\otimes 2}}[(\partial\langle\mathbf{O}\rangle)^2]$. Let us write the integral for the second moment in full, and rearrange terms appropriately:

$$\mathbb{E}_{g^+,g^-\sim\mu^{\otimes 2}}[(\partial\langle\mathbf{O}\rangle)^2] = \iint_G (\text{Tr}(\mathbf{U}_{g^-}\rho\mathbf{U}_{g^-}^\dagger[\mathbf{H}, \mathbf{U}_{g^+}\mathbf{O}\mathbf{U}_{g^+}^\dagger]))^2 dg^- dg^+ \tag{55}$$

$$= \int_G \text{Tr}\left\{\left(\int_G \mathbf{U}_g^{-\otimes 2}\rho^{\otimes 2}\mathbf{U}_g^{-\dagger\otimes 2} dg^-\right)[\mathbf{H}, \mathbf{U}_g^+\mathbf{O}\mathbf{U}_g^{+\dagger}]^{\otimes 2}\right\} dg^+. \tag{56}$$

Given that $\mathbf{O}$ is a projector onto another computational basis state, i.e. an element of V_{λ_0}, we can use our above arguments to simplify the integral as follows:

$$\mathbb{E}_{g^+,g^-\sim\mu^{\otimes 2}}[(\partial\langle\mathbf{O}\rangle)^2] = \frac{1}{\dim V_{\lambda_0}} \int_G \text{Tr}\left\{P_{\lambda_0}[\mathbf{H}, \mathbf{U}_g^+\mathbf{O}\mathbf{U}_g^{+\dagger}]^{\otimes 2}\right\} dg^+ \tag{57}$$

$$= \frac{1}{\dim V_{\lambda_0}}\text{Tr}\Big\{\int_G (\mathbf{U}_{g^+}\mathbf{O}\mathbf{U}_{g^+}^\dagger)^{\otimes 2} dg^+ [P_{\lambda_0}\mathbf{H}^{\otimes 2} - (\mathbf{H}\otimes\mathbb{1})P_{\lambda_0}(\mathbb{1}\otimes\mathbf{H})$$
$$- (\mathbb{1}\otimes\mathbf{H})P_{\lambda_0}(\mathbf{H}\otimes\mathbb{1}) + \mathbf{H}^{\otimes 2}P_{\lambda_0}]\Big\} \tag{58}$$

$$= \frac{1}{\dim V_{\lambda_0}^2}\text{Tr}\Big\{P_{\lambda_0}[P_{\lambda_0}\mathbf{H}^{\otimes 2} - (\mathbf{H}\otimes\mathbb{1})P_{\lambda_0}(\mathbb{1}\otimes\mathbf{H})$$
$$- (\mathbb{1}\otimes\mathbf{H})P_{\lambda_0}(\mathbf{H}\otimes\mathbb{1}) + \mathbf{H}^{\otimes 2}P_{\lambda_0}]\Big\} \tag{59}$$

$$= \frac{1}{\dim V_{\lambda_0}^2}\left(2\text{Tr}[P_{\lambda_0}\mathbf{H}^{\otimes 2}] - \text{Tr}[P_{\lambda_0}(\mathbf{H}\otimes\mathbb{1})P_{\lambda_0}(\mathbb{1}\otimes\mathbf{H})] - \text{Tr}[P_{\lambda_0}(\mathbb{1}\otimes\mathbf{H})P_{\lambda_0}(\mathbf{H}\otimes\mathbb{1})]\right). \tag{60}$$

One can observe that the elements of the Gelfand–Cetlin basis for V_{λ_0}, i.e. elements in $\mathrm{Im}(P_{\lambda_0})$, are invariant under any swaps across the center tensor product, i.e. row swaps on the SSYT. Furthermore, since any $\mathbf{U}_g \otimes \mathbf{U}_g$ commutes with SWAP across the center $\otimes$, we have: $P_{\lambda_0}\mathrm{SWAP} = \mathrm{SWAP}P_{\lambda_0} = P_{\lambda_0}$. This gives

$$\begin{aligned}
&\mathrm{Tr}[P_{\lambda_0}(\mathbf{H}\otimes\mathbb{1})P_{\lambda_0}(\mathbb{1}\otimes\mathbf{H})] && (61)\\
&= \mathrm{Tr}[P_{\lambda_0}(\mathbf{H}\otimes\mathbb{1})\mathrm{SWAP}P_{\lambda_0}\mathrm{SWAP}(\mathbb{1}\otimes\mathbf{H})] && (62)\\
&= \mathrm{Tr}[P_{\lambda_0}\mathrm{SWAP}(\mathbb{1}\otimes\mathbf{H})P_{\lambda_0}(\mathbf{H}\otimes\mathbb{1})\mathrm{SWAP}] && (63)\\
&= \mathrm{Tr}[P_{\lambda_0}(\mathbb{1}\otimes\mathbf{H})P_{\lambda_0}(\mathbf{H}\otimes\mathbb{1})], && (64)
\end{aligned}$$

and

$$\begin{aligned}
&\mathrm{Tr}[P_{\lambda_0}(\mathbf{H}\otimes\mathbb{1})P_{\lambda_0}(\mathbb{1}\otimes\mathbf{H})] && (65)\\
&= \mathrm{Tr}[P_{\lambda_0}(\mathbf{H}\otimes\mathbb{1})P_{\lambda_0}\mathrm{SWAP}(\mathbb{1}\otimes\mathbf{H})] && (66)\\
&= \mathrm{Tr}[P_{\lambda_0}(\mathbf{H}\otimes\mathbb{1})P_{\lambda_0}(\mathbf{H}\otimes\mathbb{1})\mathrm{SWAP}] && (67)\\
&= \mathrm{Tr}[P_{\lambda_0}(\mathbf{H}\otimes\mathbb{1})P_{\lambda_0}(\mathbf{H}\otimes\mathbb{1})]. && (68)
\end{aligned}$$

Note that $\tilde{\mathbf{H}} := \mathbb{1}\otimes\mathbf{H}+\mathbf{H}\otimes\mathbb{1}$ commutes with P_{λ_0} since $\phi(\mathrm{SU}(n))$ commutes with P_{λ_0} and $\tilde{\mathbf{H}} \in d\phi(\mathfrak{su}(n))\otimes d\phi(\mathfrak{su}(n))$. All of the above results imply that:

$$\begin{aligned}
&\mathrm{Tr}[P_{\lambda_0}(\mathbb{1}\otimes\mathbf{H}^2+\mathbf{H}^2\otimes\mathbb{1})] + 2\mathrm{Tr}[P_{\lambda_0}\mathbf{H}^{\otimes 2}] && (69)\\
&= \mathrm{Tr}[P_{\lambda_0}(\mathbb{1}\otimes\mathbf{H}+\mathbf{H}\otimes\mathbb{1})^2] && (70)\\
&= \mathrm{Tr}[P_{\lambda_0}(\mathbb{1}\otimes\mathbf{H}+\mathbf{H}\otimes\mathbb{1})P_{\lambda_0}(\mathbb{1}\otimes\mathbf{H}+\mathbf{H}\otimes\mathbb{1})] && (71)\\
&= \mathrm{Tr}[P_{\lambda_0}(\mathbf{H}\otimes\mathbb{1})P_{\lambda_0}(\mathbf{H}\otimes\mathbb{1})] + \mathrm{Tr}[P_{\lambda_0}(\mathbf{H}\otimes\mathbb{1})P_{\lambda_0}(\mathbb{1}\otimes\mathbf{H})] && (72)\\
&\quad + \mathrm{Tr}[P_{\lambda_0}(\mathbb{1}\otimes\mathbf{H})P_{\lambda_0}(\mathbf{H}\otimes\mathbb{1})] + \mathrm{Tr}[P_{\lambda_0}(\mathbb{1}\otimes\mathbf{H})P_{\lambda_0}(\mathbb{1}\otimes\mathbf{H})] && (73)\\
&= 4\mathrm{Tr}[P_{\lambda_0}(\mathbf{H}\otimes\mathbb{1})P_{\lambda_0}(\mathbf{H}\otimes\mathbb{1})], && (74)
\end{aligned}$$

which implies that

$$2\mathrm{Tr}[P_{\lambda_0}\mathbf{H}^{\otimes 2}] - 2\mathrm{Tr}[P_{\lambda}(\mathbf{H}\otimes\mathbb{1})P_{\lambda_0}(\mathbf{H}\otimes\mathbb{1})] = 2\mathrm{Tr}[P_{\lambda_0}(\mathbf{H}\otimes\mathbb{1})P_{\lambda_0}(\mathbf{H}\otimes\mathbb{1})] - \mathrm{Tr}[P_{\lambda_0}(\mathbb{1}\otimes\mathbf{H}^2+\mathbf{H}^2\otimes\mathbb{1})]. \quad (75)$$

Since $\mathbf{H}$ is skew-Hermitian and P_{λ_0} is Hermitian, we have that $-\mathbf{H}^2 \succcurlyeq 0$, and

$$-\mathrm{Tr}[P_{\lambda_0}(\mathbf{H}\otimes\mathbb{1})P_{\lambda_0}(\mathbf{H}\otimes\mathbb{1})] = \|P_{\lambda_0}(\mathbf{H}\otimes\mathbb{1})\|_F^2. \quad (76)$$

Thus,

$$\begin{aligned}
\mathbb{E}_{g^+,g^-\sim\mu^{\otimes 2}}[(\partial\langle\mathbf{O}\rangle)^2] &= \frac{2}{\dim V_{\lambda_0}^2}\left(\mathrm{Tr}[P_{\lambda_0}\mathbf{H}^{\otimes 2}] - \mathrm{Tr}[P_{\lambda_0}(\mathbf{H}\otimes\mathbb{1})P_{\lambda_0}(\mathbf{H}\otimes\mathbb{1})]\right) && (77)\\
&= \frac{1}{\dim V_{\lambda_0}^2}(2\mathrm{Tr}[P_{\lambda}(\mathbf{H}\otimes\mathbb{1})P_{\lambda_0}(\mathbf{H}\otimes\mathbb{1})] - \mathrm{Tr}[P_{\lambda_0}(\mathbb{1}\otimes\mathbf{H}^2+\mathbf{H}^2\otimes\mathbb{1})]) && (78)\\
&= \frac{1}{\dim V_{\lambda_0}^2}(\mathrm{Tr}[P_{\lambda_0}(\mathbb{1}\otimes -\mathbf{H}^2 + -\mathbf{H}^2\otimes\mathbb{1})] - 2\|P_{\lambda_0}(\mathbf{H}\otimes\mathbb{1})\|_F^2) && (79)\\
&= \frac{2}{\dim V_{\lambda_0}^2}(\mathrm{Tr}[P_{\lambda_0}(\mathbb{1}\otimes -\mathbf{H}^2)P_{\lambda_0}] - \|P_{\lambda_0}(\mathbf{H}\otimes\mathbb{1})\|_F^2) && (80)\\
&\leq \frac{2}{\dim V_{\lambda_0}^2}\mathrm{Tr}[P_{\lambda_0}(\mathbb{1}\otimes -\mathbf{H}^2)P_{\lambda_0}] && (81)\\
&\leq \frac{2\|\mathbf{H}\|_2^2}{\dim V_{\lambda_0}}, && (82)
\end{aligned}$$

where the fourth equality follows from invariance of P_{λ_0} under SWAP, and the last inequality follows from $\mathbb{1}\otimes -\mathbf{H}^2 \succcurlyeq 0$.

Since $\mathbf{H}$ is effectively the restriction of one of the quantum compound ansatz generators to the Hamming-weight $n/2$ subspace, the spectral norm is constant. Thus

$$\mathbb{E}_{g^+,g^-\sim\mu^{\otimes 2}}[(\partial\langle\mathbf{O}\rangle)^2] \in \mathcal{O}(1/\dim V_{\lambda_0}). \quad (83)$$

The result follows by plugging in the result of Supplementary Lemma 4. □

Supplementary Note 5 – Projected Norm Lower Bound

There is actually another interpretation of the projected norm $\|\rho_\alpha\|_{\mathrm{F}}^2$ in terms of a different norm that has a deeper connection to the simple ideals $\mathfrak{g}_\alpha$ and leads to a generic lower bound on $\|\rho_\alpha\|_{\mathrm{F}}^2$. This section makes use of the representation theory of semisimple Lie algebra (see Supplementary Note 1 for an introduction).

Let the set Δ^+ will denote the collection of positive simple roots. In addition if T_{ij} is the metric tensor for the Killing form, then for roots $\vec{\alpha}, \vec{\beta} \in \Delta^+$ we define the inner product (recall that the Killing form is positive definite when restricted to the Cartan sublagebra for semisimple Lie algebra):

$$(\vec{\alpha}, \vec{\beta})_{\mathrm{w}} := [T^{-1}]_{ij}\alpha_i\beta_j, \tag{84}$$

which linearly extends to weights expressed in terms of Δ^+. The induced norm will be denoted $\|\cdot\|_{\mathrm{w}}$.

The following lemma characterizes the action of the split Casimir on weight vectors. While potentially already a well-known result, we could not find an existing reference. Thus we include a short proof for completeness, which is a simple computation.

Supplementary Lemma 5. *Let $\mathfrak{g}$ be a simple Lie algebra. Suppose V is a finite-dimensional inner product space and $d\phi : \mathfrak{g} \to \mathfrak{u}(V)$ is a representation of $\mathfrak{g}$. If $|\vec{\lambda}\rangle \in V$ is a weight vector with weight $\vec{\lambda}$ and $\mathbf{K}$ is the normalized split Casimir, then*

$$\langle\vec{\lambda}|^{\otimes 2}\mathbf{K}|\vec{\lambda}\rangle^{\otimes 2} = \frac{\|\vec{\lambda}\|_w^2}{I_\phi}. \tag{85}$$

Proof. Let us the denote the Cartan–Weyl basis for the complexification of $\mathfrak{g}$ by:

$$\{H_i\}_{i=1}^r \cup \{E_{\vec{\alpha}}, E_{-\vec{\alpha}}\}_{\vec{\alpha}\in\Delta^+}, \tag{86}$$

where Δ^+ is a set of positive simple roots, the H_i span the Cartan subalgebra and $E_{\vec{\alpha}}, E_{-\vec{\alpha}}$ are the ladder operators. In addition, let $d\phi_{\mathbb{C}}$ denote the complexification of $d\phi$.

We can express the normalized split Casimir in the Cartan–Weyl basis as:

$$\mathbf{K} = I_\phi^{-1}\left(\sum_{i=1}^r d\phi_{\mathbb{C}}(H_i)\otimes d\phi_{\mathbb{C}}(H_i) + \sum_{\vec{\alpha}\in\Delta^+}\left[d\phi_{\mathbb{C}}(E_{\vec{\alpha}})\otimes d\phi_{\mathbb{C}}(E_{-\vec{\alpha}}) + d\phi_{\mathbb{C}}(E_{-\vec{\alpha}})\otimes d\phi_{\mathbb{C}}(E_{\vec{\alpha}})\right]\right). \tag{87}$$

$$\langle\vec{\lambda}|^{\otimes 2}\mathbf{K}|\vec{\lambda}\rangle^{\otimes 2} = I_\phi^{-1}\left(\sum_{i=1}^r(\langle\vec{\lambda}|d\phi_{\mathbb{C}}(H_i)|\vec{\lambda}\rangle)^2 + \sum_{\vec{\alpha}\in\Delta^+}2\langle\vec{\lambda}|d\phi_{\mathbb{C}}(E_{\vec{\alpha}})|\vec{\lambda}\rangle\langle\vec{\lambda}|d\phi_{\mathbb{C}}(E_{-\vec{\alpha}})|\vec{\lambda}\rangle\right) \tag{88}$$

$$\tag{89}$$

$$= I_\phi^{-1}\left(\sum_{i=1}^r\lambda_i^2 + \sum_{\vec{\alpha}\in\Delta^+}2\langle\vec{\lambda}|d\phi_{\mathbb{C}}(E_{\vec{\alpha}})|\vec{\lambda}\rangle\langle\vec{\lambda}|d\phi_{\mathbb{C}}(E_{-\vec{\alpha}})|\vec{\lambda}\rangle\right) \tag{90}$$

$$= I_\phi^{-1}\left(\sum_{i=1}^r\lambda_i^2\right) \tag{91}$$

$$= \frac{\|\vec{\lambda}\|_{\mathrm{w}}^2}{I_\phi}, \tag{92}$$

where $d\phi_{\mathbb{C}}(E_{\pm\vec{\alpha}})$ is zero because the $E_{\pm\vec{\alpha}}$ move between orthogonal weight spaces. □

Suppose $\rho = |\psi\rangle\langle\psi|$ for some unit vector $|\psi\rangle \in V$, which corresponds to the pure state case for quantum. If the representation under consideration ϕ is not irreducible, then, by unitarity, we can decompose ϕ into orthogonal irreducible components:

$$V = \bigoplus_r V_{\phi_r}. \tag{93}$$

Furthermore, each irreducible V_{ϕ_r} can be represented as a direct sum of, mutually orthogonal, weight spaces $V_{\vec{\lambda}^{(k)}}$ for weight $\vec{\lambda}^{(k)}$:

$$V_{\phi_r} = \bigoplus_t V_{\vec{\lambda}^{(t)}}. \tag{94}$$

Thus $|\psi\rangle \in V$ can be uniquely expressed as a linear combination of weight vectors:

$$|\psi\rangle = \sum_k \beta_k |\vec{\lambda}^{(k)}\rangle. \tag{95}$$

To every unit vector $|\psi\rangle \in V$ we can associate the following vector:

$$\vec{\psi} = \sum_k |\beta_k|^2 \vec{\lambda}^{(k)}, \tag{96}$$

which by the unit vector assumption is a convex combination of weights. Note that a generalization to non-unit vectors follows trivially, i.e. $c|\psi\rangle \implies c^2\vec{\psi}$. Since V is a complex vector space, a single $\vec{\psi}$ can be associated with multiple unit vectors.

We can linearly extend the inner product for weights, $(\cdot,\cdot)_{\mathrm{w}}$, to the quantity in Supplementary Equation (96) to obtain the norm:

$$\|\vec{\psi}\|_{\mathrm{w}}^2 = \sum_{k,j} |\beta_k|^2 |\beta_j|^2 (\vec{\lambda}^{(k)}, \vec{\lambda}^{(j)})_{\mathrm{w}}. \tag{97}$$

This is the quantity that lower bounds the projected norm, as put concretely in the following result.

Supplementary Theorem 2 (Projected Norm Lower Bound)**.** *Suppose ϕ is unitary representation of a simple Lie algebra $\mathfrak{g}$, then for any unit vector $|\psi\rangle \in V$ the following holds*

$$\|\rho_{\mathfrak{g}}\|_{\mathrm{F}} \geq \frac{\|\vec{\psi}\|_{\mathrm{w}}^2}{I_\phi}, \tag{98}$$

where $\rho = |\psi\rangle\langle\psi|$.

Proof. We know that by definition $\|P_{\mathfrak{g}}|\psi\rangle\langle\psi|\|_{\mathrm{F}} = \langle\psi|^{\otimes 2}\mathbf{K}|\psi\rangle^{\otimes 2}$. Using the expression for the normalized split Casimir in the Cartan–Weyl basis:

$$\langle\psi|^{\otimes 2}\mathbf{K}|\psi\rangle^{\otimes 2} = I_\phi^{-1}\left(\sum_{i=1}^r (\langle\psi|d\phi_{\mathbb{C}}(H_i)|\psi\rangle)^2 + \sum_{\vec{\alpha}\in\Delta^+} 2\langle\psi|d\phi_{\mathbb{C}}(E_{\vec{\alpha}})|\psi\rangle\langle\psi|d\phi_{\mathbb{C}}(E_{-\vec{\alpha}})|\psi\rangle\right). \tag{99}$$

Note that

$$\sum_{\vec{\alpha}\in\Delta^+} \langle\psi|^{\otimes 2} d\phi_{\mathbb{C}}(E_{\vec{\alpha}}) \otimes d\phi_{\mathbb{C}}(E_{-\vec{\alpha}})|\psi\rangle^{\otimes 2} = \sum_{\vec{\alpha}\in\Delta^+} \langle\psi|d\phi_{\mathbb{C}}(E_{\vec{\alpha}})|\psi\rangle\langle\psi|d\phi_{\mathbb{C}}(E_{-\vec{\alpha}})|\psi\rangle \tag{100}$$

$$= \sum_{\vec{\alpha}\in\Delta^+} \langle\psi|d\phi_{\mathbb{C}}(E_{\vec{\alpha}})|\psi\rangle\langle\psi|d\phi_{\mathbb{C}}(E_{\vec{\alpha}})^\dagger|\psi\rangle \tag{101}$$

$$= \sum_{\vec{\alpha}\in\Delta^+} |\langle\psi|d\phi_{\mathbb{C}}(E_{\vec{\alpha}})|\psi\rangle|^2 \geq 0 \tag{102}$$

since by the unitarity of the representation: $d\phi_{\mathbb{C}}(E_{-\vec{\alpha}}) = d\phi_{\mathbb{C}}(E_{\vec{\alpha}})^\dagger$. Therefore:

$$\langle\psi|^{\otimes 2}\mathbf{K}|\psi\rangle^{\otimes 2} \geq I_\phi^{-1} \sum_{kr} |\beta_k|^2 |\beta_r|^2 \sum_{i=1}^r \vec{\lambda}^{(k)}(d\phi_{\mathbb{C}}(H_i)) \cdot \vec{\lambda}^{(r)}(d\phi_{\mathbb{C}}(H_i)) \tag{103}$$

$$= I_\phi^{-1} \sum_{kr} |\beta_k|^2 |\beta_r|^2 (\vec{\lambda}^{(k)}, \vec{\lambda}^{(r)})_{\mathrm{w}} \tag{104}$$

$$= \frac{\|\psi\|_{\mathrm{w}}^2}{I_\phi}. \tag{105}$$

□

Note that $\sum_{\vec{\alpha}} |\langle\psi|d\phi_{\mathbb{C}}(E_{\vec{\alpha}})|\psi\rangle|^2 > 0$ if and only if the vector has support on at least two weight spaces where the weights differ by a root $\vec{\alpha}$. Thus we have equality, for example, if $|\psi\rangle = |\vec{\lambda}^{(k)}\rangle$ for some weight $\vec{\lambda}^{(k)}$, however there can be weights that differ by multiples of a root, which by definition is not a root, so this is not a necessary condition.

We can use Supplementary Theorem 2 to give a lower bound on the variance when the initial state ρ is pure:

$$\mathrm{GradVar} \geq \frac{I_{\mathrm{Ad}}\|o\|_{\mathfrak{g}}^2\|h\|_{\mathfrak{g}}^2}{d_{\mathfrak{g}}^2} \cdot \|\vec{\psi}\|_{\mathrm{w}}^2, \tag{106}$$

which helps to remove the dependence on the index I_ϕ. Note that the only ϕ-dependent quantity is $\|\vec{\psi}\|_{\mathrm{w}}^2$. In the semisimple case, i.e. arbitray LASA, the above result can be applied separately to each simple ideal to yield:

$$\mathrm{GradVar} \geq \sum_{\alpha} \frac{I_{\mathrm{Ad}_\alpha}\|o\|_{\mathfrak{g}_\alpha}^2\|h\|_{\mathfrak{g}_\alpha}^2}{d_{\mathfrak{g}_\alpha}^2} \cdot \|\vec{\psi}\|_{\mathrm{w}_\alpha}^2, \tag{107}$$

where the subscript α is added to the norm $\|\cdot\|_{\mathrm{w}}$ to emphasize it is different for each ideal. Specifically, the norm depends on the restriction of $d\phi$ to $\mathfrak{g}_\alpha$, which may break V into a different set of irreducible components for different α. Since $I_{\mathrm{Ad}} = \Theta(\sqrt{d_{\mathfrak{g}_\alpha}})$, this implies that

$$\mathrm{GradVar} \in \Omega\left(\sum_{\alpha} \frac{\|o\|_{\mathfrak{g}_\alpha}^2\|h\|_{\mathfrak{g}_\alpha}^2\|\vec{\psi}\|_{\mathrm{w}_\alpha}^2}{d_{\mathfrak{g}_\alpha}^{3/2}}\right). \tag{108}$$

One can contrast this with the upper bound presented in the main text where $\mathbf{O}$ and $\mathbf{H}$ had ϕ-dependent norms, i.e. were Frobenius norms. Alternatively, the above lower bound has shifted all the ϕ dependence to the $\|\vec{\psi}\|_{\mathrm{w}_\alpha}^2$ norms.

If $\mathbf{O}$ and $\mathbf{H}$ are chosen such that they have mutual alignment on $\mathfrak{g}_\alpha$'s that do not have an exponentially growing dimension, then Supplementary Equation (108) gives more insight into how the initial state needs to be chosen to avoid a BP. In this setting, one can view the terms

$$\frac{\|o\|_{\mathfrak{g}_\alpha}^2\|h\|_{\mathfrak{g}_\alpha}^2}{d_{\mathfrak{g}_\alpha}^{3/2}} \tag{109}$$

as coefficients that weigh the different norms $\|\vec{\psi}\|_{\mathrm{w}_\alpha}^2$. Thus, the goal is to select an initial state such that $\|\vec{\psi}\|_{\mathrm{w}_\alpha}^2$ are decaying slowly on the subalgebras which $\mathbf{O}$ and $\mathbf{H}$ agree on. A poor choice of initial state can cause the variance to fall faster than the DLA dimension. Unfortunately, the $\|\vec{\psi}\|_{\mathrm{w}_\alpha}^2$ can be challenging to determine in practice, even at small scales. Thus, at the moment we consider the lower bound to be more of theoretical interest and use it to further enlighten the BP phenomenon in LASA.

Supplementary Note 6 – Applicability of Theory beyond LASA

In this section, we present generalizations of the Theorem 2.9 in the main text to the non-LASA case. Specifically, the same tools utilized to derive the results for LASA can be used to obtain a lower bound on gradient variance for an arbitrary observable, in terms of the LASA component. Unfortunately, the expression is not as concise as the ones in the main text, and the various factors that contribute to a BP can be challenging to compute. However, there are still some interesting observations that can be made. For clarity, the proofs of the results of this section have been delayed to a separate subsection.

Let us denote the orthogonal complement, under the standard Frobenius inner product, of $d\phi(\mathfrak{g})$ within $\mathfrak{u}(V)$ as $(d\phi(\mathfrak{g}))^c$. Then we can decompose an arbitrary element $\mathbf{O} \in \mathfrak{u}(V)$ (a generic skew-Hermitian operator on V) as

$$\mathbf{O} = \mathbf{O}_{\mathfrak{g}} + \mathbf{O}_{\mathfrak{g}^c}, \tag{110}$$

where $\mathbf{O}_{\mathfrak{g}} \in d\phi(\mathfrak{g})$ and $\mathbf{O}_{\mathfrak{g}^c} \in (d\phi(\mathfrak{g}))^c$. Via conjugation, ϕ induces an action of G on the whole of $\mathfrak{u}(V)$ (*a real unitary representation*), which breaks up into two subrepresentations. One is the usual adjoint representation Ad_G on $d\phi(\mathfrak{g})$, and the other is $\phi(G)$ acting on $(d\phi(\mathfrak{g}))^c$ via conjugation. The orthogonal complement is also an invariant subspace. Since G is compact, both $d\phi(\mathfrak{g})$ and its complement will decompose into a direct sum of irreducible components. Thus overall,

$$\mathfrak{u}(V) = \bigoplus_{\kappa} W_\kappa, \tag{111}$$

where each W_κ is an irreducible subspace over $\mathbb{R}$ under conjugation by $\phi(G)$.

The space $d\phi(\mathfrak{g})$ breaks into a direct sum of simple ideals, where all methods from the main text apply. Unfortunately, it is not possible to make general statements about the decomposition of the complement besides that there may be an irreducible component in $(d\phi(\mathfrak{g}))^c$ that is G-isomorphic to one of the simple ideals of $\mathfrak{g}$.

For an observable that has support on the complement, the cost function will generally split into three terms: the variance on $d\phi(\mathfrak{g})$, the variance on $(d\phi(\mathfrak{g}))^c$, and an interaction term (the covariance). Explicitly, it is the following sum:

$$\text{GradVar} = \text{Var}_{(g_+,g_-)\sim\mu^{\otimes 2}}[\partial\langle \mathbf{O}_{\mathfrak{g}}\rangle_\rho] + \text{Var}_{(g_+,g_-)\sim\mu^{\otimes 2}}[\partial\langle \mathbf{O}_{\mathfrak{g}^c}\rangle_\rho] \tag{112}$$

$$+ 2\text{Cov}_{(g_+,g_-)\sim\mu^{\otimes 2}}[\partial\langle \mathbf{O}_{\mathfrak{g}}\rangle_\rho, \partial\langle \mathbf{O}_{\mathfrak{g}^c}\rangle_\rho]. \tag{113}$$

Interestingly, for the covariance terms, all that matters is whether there is an irreducible component, W_κ, of $(d\phi(\mathfrak{g}))^c$ that is G-isomorphic to a simple ideal of $\mathfrak{g}$, i.e. isomorphic to an irreducible component of $d\phi(\mathfrak{g})$. Furthermore, since each of the simple ideals are non-isomorphic, there is only one cross term per W_κ. Both of the previous statements follow from Schur orthogonality, which will cause cross terms involving non-isomorphic irreducible representations to be annihilated. Thus, we can further split a general observable into three components

$$\mathbf{O} = \mathbf{O}_{\mathfrak{g}} + \mathbf{O}_{\mathfrak{g}^c_{\cong}} + \mathbf{O}_{\mathfrak{g}^c_{\ncong}}, \tag{114}$$

where $\mathbf{O}_{\mathfrak{g}^c_{\cong}}$ denotes the sum of components that are in an irreducible component that is G-isomorphic to a simple ideal and $\mathbf{O}_{\mathfrak{g}^c_{\ncong}}$ is the sum of those that are not. We will term the former as the *ideal complement* component and the later as the *non-ideal complement* component. We will call $\mathbf{O}_{\mathfrak{g}}$ the *ideal* component. So in Supplementary Equation (111) each W_κ is either part of the ideal, ideal complement or non-ideal complement components. For two indices κ, κ', the notation $\kappa \cong \kappa'$ will imply that W_κ is G-isomorphic to $W_{\kappa'}$. Lastly, note that depending on the observable and Lie algebra, the ideal or non-ideal complement components could be empty.

As just discussed, Schur orthogonality allows us to express the general variance as

$$\text{GradVar} = \text{Var}_{(g_+,g_-)\sim\mu_\alpha^{\otimes 2}}[\partial\langle \mathbf{O}_{\mathfrak{g}}\rangle_\rho] \tag{115}$$

$$+ \text{Var}_{(g_+,g_-)\sim\mu^{\otimes 2}}[\partial\langle \mathbf{O}_{\mathfrak{g}^c_{\cong}}\rangle_\rho] \tag{116}$$

$$+ \text{Var}_{(g_+,g_-)\sim\mu^{\otimes 2}}[\partial\langle \mathbf{O}_{\mathfrak{g}^c_{\ncong}}\rangle_\rho] \tag{117}$$

$$+ 2\text{Cov}_{(g_+,g_-)\sim\mu^{\otimes 2}}[\partial\langle \mathbf{O}_{\mathfrak{g}}\rangle_\rho, \partial\langle \mathbf{O}_{\mathfrak{g}^c_{\cong}}\rangle_\rho]. \tag{118}$$

The lower bound on the general variance that we present is in terms of the ideal and ideal complement components of Supplementary Equation (115), which together we call the *DLA component* of the variance. Before proceeding with the result, we start with defining two new quantities that will appear in the lower bound.

Supplementary Definition 3. *Suppose W_1 and W_2 are G-isomorphic irreducible components of $\mathfrak{u}(V)$, with an arbitrary G-isomorphism $\gamma : W_2 \to W_1$. Let $i\mathbf{B}, i\mathbf{C} \in \mathfrak{u}(V)$, and let $i\mathbf{B}_1$ ($i\mathbf{B}_2$) and $i\mathbf{C}_1$ ($i\mathbf{C}_2$) denote their orthogonal projections (under the Frobenius inner product) onto W_1 (W_2). Then we define:*

$$(\mathbf{B}, \mathbf{C})_{1,2} = \text{Tr}(\mathbf{B}_1\gamma\mathbf{B}_2)\text{Tr}(\mathbf{C}_1(\gamma^{-1})^\dagger\mathbf{C}_2), \tag{119}$$

and it is independent of γ.

Intuitively, $(\mathbf{B}, \mathbf{C})_{1,2}$ is a type of "product of autocorrelations" of $\mathbf{B}$ and $\mathbf{C}$ w.r.t. their projections onto the two irreducible components.

The next quantity we define is similar to the previous one but will generalize the Killing norm quantity.

Supplementary Definition 4. *Suppose W_1 and W_2 are G-isomorphic irreducible components of $\mathfrak{u}(V)$, with an arbitrary G-isomorphism $\gamma : W_2 \to W_1$. Let $i\mathbf{A} \in \mathfrak{u}(V)$, and P_1, P_2 be orthogonal projectors onto W_1 and W_2 respectively. Then, for arbitrary orthonormal bases $\{u_j\}$ and $\{v_k\}$ of W_1 and W_2, respectively, we define:*

$$(\mathbf{A})_{1,2} := \sum_{j,k} -\langle u_j, (\gamma^{-1})^\dagger v_k\rangle \text{Tr}(P_1([\mathbf{A}, u_j])\gamma P_2([\mathbf{A}, v_k])_2), \tag{120}$$

and it is independent of γ.

If one takes $\mathbf{A}$ to be such that $\mathbf{A} \in d\phi(\mathfrak{g})$, then the operator $\mathbf{O} \mapsto [\mathbf{A}, \mathbf{O}]$ preserves the subspaces W_1 and W_2, so there is no need for the projectors P_1 and P_2. If we had $W_1 = W_2 = d\phi(\mathfrak{g}_\alpha)$, then γ would be the identity and the above quantity would be the Killing norm.

Since the variance of the complement must also be positive, we can ignore it, which leads to the following lower bound on the variance for general observables $\mathbf{O}$.

Supplementary Theorem 5 (Lower Bound by DLA component). *Let* $\mathbf{O}$ *be an arbitrary observable, then*

$$\mathrm{GradVar} \geq \sum_{\alpha} \frac{1}{d_{\mathfrak{g}_\alpha}^2} (\|\mathbf{H}_{\mathfrak{g}_\alpha}\|_{\mathrm{K}}^2 \|\mathbf{O}_{\mathfrak{g}_\alpha}\|_{\mathrm{F}}^2 \|\rho_{\mathfrak{g}_\alpha}\|_{\mathrm{F}}^2 \tag{121}$$

$$+ \sum_{\kappa|\kappa\cong\alpha} (\|\mathbf{O}_\kappa\|_{\mathrm{F}}^2 \|\rho_\kappa\|_{\mathrm{F}}^2 (i\mathbf{H})_{(\kappa,\kappa)} \tag{122}$$

$$+ \sum_{\{\kappa'|\kappa'\cong\kappa\}\cup\{\alpha\}} (\mathbf{O},\rho)_{(\kappa,\kappa')} (i\mathbf{H})_{(\kappa,\kappa')})), \tag{123}$$

where α *indexes the ideal component and* κ *indexes the ideal complement component.*

While this quantity can seem daunting, the reason for presenting it is to highlight that the decay of the variance with the DLA dimension can still appear for observables outside of the DLA, and that the techniques used to obtain the gradient variance for LASA in the previous subsection actually apply more generally. This quantity also reveals the full extent to which the size of the DLA plays a role in the gradient variance, as the non-ideal complement has no dependence on it.

If one can ensure that $\mathbf{O}_{\mathfrak{g}^c}$ has no support on W_κ isomorphic to some ideal, then one has a lower bound on the variance given completely by the ideal component, using the results for LASA. The proof of Supplementary Theorem 5 highlights that the covariance between $d\phi(\mathfrak{g})$ and its complement is

$$\mathrm{Cov}(\mathfrak{g},\mathfrak{g}^c) = \sum_{\alpha}\sum_{\kappa\cong\alpha} \frac{(\mathbf{O},\rho)_{(\kappa,\alpha)}(i\mathbf{H})_{(\kappa,\alpha)}}{d_{\mathfrak{g}_\alpha}^2}. \tag{124}$$

Unfortunately, cases where there is support on W_κ isomorphic to some ideal can easily occur. For example, take a 2-qubit system, and consider the subgroup SU(2) acting on the first qubit. Then take as operator $\mathbf{O} = \sigma_x \otimes \mathbb{1} - \sigma_x \otimes \sigma_z$, which has $i\sigma_x \otimes \mathbb{1} \in d\phi(\mathfrak{g})$ and $i\sigma_x \otimes \sigma_z \in (d\phi(\mathfrak{g}))^c$. If we take the state $|00\rangle$, since the group acts only on the first qubit, the second qubit gives expectation zero always, so that for any group element g

$$\langle \mathbf{O} \rangle_g = \langle 00|\mathbf{U}_g \sigma_x \otimes (\mathbb{1} - \sigma_z)\mathbf{U}_g^\dagger|00\rangle \tag{125}$$

$$= \langle 0|\mathbf{U}_g \sigma_x \mathbf{U}_g^\dagger|0\rangle\langle 0|(\mathbb{1} - \sigma_z)|0\rangle = 0. \tag{126}$$

Since the cost function is zero for all elements of the group, the variance must be zero. Also note that this barren plateau is not caused by the state, since not having the second qubit would give a nonzero variance.

We note that Supplementary Theorem 5 can actually be extended to a full expression of the gradient variance for an arbitrary observable. In general, this will consist of contributions from the ideal, ideal complement and non-ideal complement components. However, the contributions from the non-ideal complement can be challenging to interpret and there are a few technical caveats that need to be addressed. We have placed the more general result in Supplementary Note 6 B.

A. Proof of Supplementary Theorem 5

We start this section by showing that the quantity from Supplementary Definitions 3 and 4 are actually well-defined, specifically that they is independent of the choice of G-isomorphism γ. We will do this by looking at a generalized version of these quantities and show how it comes up when computing inner products of matrix coefficients. This is done by using Schur orthogonality for isomorphic yet not equal representations (the following lemma). We were unable to find this result in literature, and thus we have included a proof for completeness.

Supplementary Lemma 6. *Suppose* $\phi : G \to \mathcal{U}(V)$ *is a unitary representation of* G*, and* V_1, V_2 *are two isomorphic subrepresentations of* V*. Let* $\gamma : V_2 \to V_1$ *be an arbitrary* G*-isomorphism. In addition, we assume that either of the following two conditions is satisfied:*

- *if* ψ *is over* $\mathbb{C}$*, then* V_1 *and* V_2 *are irreducible,*
- *or if* ψ *is over* $\mathbb{R}$*, then* V_1 *and* V_2 *are irreducible and their complexifications are irreducible.*

Then, in orthonormal bases, the following holds for the matrix coefficients:

$$\dim V_1 \int_G \phi_{j_1,j_2}^{(1)}(g)\overline{\phi_{k_1,k_2}^{(2)}(g)}dg = \langle \gamma v_{k_2}, u_{j_2}\rangle\langle \gamma^{-1}u_{j_1}, v_{k_1}\rangle. \tag{127}$$

Proof. The proof of the lemma is a modification of the standard Schur orthogonality proof found in Ref. [1, Corollary 4.10]. Let $\{u_j\}$ and $\{v_k\}$ denote orthonormal bases for V_1 and V_2, respectively. In addition, $\phi^{(1)}$ and $\phi^{(2)}$ are the corresponding subrepresentations. We define a linear operator $B_{a,b} : V_1 \to V_2$ as $B_{a,b}x := \langle x, u_b\rangle v_a$ for $x \in V_1$. We can relate the inner product of matrix coefficients to this operator $B_{a,b}$:

$$\int_G \phi^{(1)}_{j_1,j_2}(g)\overline{\phi^{(2)}_{k_1,k_2}(g)}dg = \int_G \langle\phi^{(1)}(g)u_{j_1}, u_{j_2}\rangle\langle\phi^{(2)}(g^{-1})v_{k_2}, v_{k_1}\rangle dg \tag{128}$$

$$= \langle\int_G \langle\phi^{(1)}(g)u_{j_1}, u_{j_2}\rangle \cdot \phi^{(2)}(g^{-1})v_{k_2}dg, v_{k_1}\rangle \tag{129}$$

$$= \langle\left[\int_G \phi^{(2)}(g^{-1})B_{j_2,k_2}\phi^{(1)}(g)dg\right] u_{j_1}, v_{k_1}\rangle \tag{130}$$

$$= \langle\mathcal{A}(B_{j_2,k_2})u_{j_1}, v_{k_1}\rangle, \tag{131}$$

where for any linear operator B, $\mathcal{A}(B)$ is called the twirling operator:

$$\mathcal{A}(B) = \int_G \phi^{(2)}(g^{-1})B\phi^{(1)}(g)dg. \tag{132}$$

The properties of the Haar measure imply that $\mathcal{A}(B)$ is G-equivariant for any B. Thus the composition of $\mathcal{A}(B)$ and γ:

$$\gamma\mathcal{A}(B) : V_1 \to V_1 \tag{133}$$

is G-equivariant.

The hypothesis of the lemma allows us to apply Schur's lemma regardless if the representation complex or real. Specifically, $\gamma\mathcal{A}(B_{j_2,k_2}) = \lambda_\gamma \mathbb{1}$, where λ_γ is complex or real depending on which ψ is. In addition, any G-isomorphism of V_1 and V_2 is a scalar (in the same field that ψ is over) multiple of γ.

Thus,

$$\int_G \phi^{(1)}_{j_1,j_2}(g)\overline{\phi^{(2)}_{k_1,k_2}(g)}dg = \langle\mathcal{A}(B_{j_2,k_2})u_{j_1}, v_{k_1}\rangle \tag{134}$$

$$= \langle\gamma^{-1}\gamma\mathcal{A}(B_{j_2,k_2})u_{j_1}, v_{k_1}\rangle \tag{135}$$

$$= \lambda_\gamma\langle\gamma^{-1}u_{j_1}, v_{k_1}\rangle. \tag{136}$$

Let $n := \dim V_{\psi^{(1)}} = \dim V_{\psi^{(2)}}$. We can use the following to solve for λ_γ:

$$\lambda_\gamma n = \mathrm{Tr}(\gamma A(B_{j_2,k_2})) \tag{137}$$

$$= \int_G \sum_{j=1}^{n} \langle\gamma\phi^{(2)}(g^{-1})B_{j_2,k_2}\phi^{(1)}(g)u_j, u_j\rangle dg \tag{138}$$

$$= \int_G \sum_{j=1}^{n} \langle\phi^{(1)}(g^{-1})\gamma B_{j_2,k_2}\phi^{(1)}(g)u_j, u_j\rangle dg \tag{139}$$

$$= \int_G \sum_{j=1}^{n} \langle\gamma B_{j_2,k_2}\phi^{(1)}(g)u_j, \phi^{(1)}(g)u_j\rangle dg \tag{140}$$

$$= \mathrm{Tr}(\gamma B_{j_2,k_2}), \tag{141}$$

where for $\gamma B_{j_2,k_2}x = \langle x, u_{j_2}\rangle\gamma v_{k_2}$ we have $\mathrm{Tr}(\gamma B_{j_2,k_2}) = \langle\gamma v_{k_2}, u_{j_2}\rangle$. Solving for λ_γ gives:

$$\lambda_\gamma = \frac{\langle\gamma v_{k_2}, u_{j_2}\rangle}{n}. \tag{142}$$

We can plug this result back in to obtain an expression for the inner product of matrix coefficients

$$\int_G \phi^{(1)}_{j_1,j_2}(g)\overline{\phi^{(2)}_{k_1,k_2}(g)}dg = \frac{\langle\gamma v_{k_2}, u_{j_2}\rangle\langle\gamma^{-1}u_{j_1}, v_{k_1}\rangle}{n}. \tag{143}$$

The γ-independence of the left-hand side follows from the γ-independence of the right-hand side or by noting that any two G-equivariant maps will be scalar multiples of each other. □

The following lemma uses the previous result to generalize Lemma 4.2.

Supplementary Lemma 7. *Suppose G is a compact matrix Lie group with Lie algebra $\mathfrak{g} \subseteq \mathfrak{u}(m)$, where $\mathfrak{u}(m)$ is the Lie algebra of $m \times m$ skew-Hermitian matrices. Suppose $\psi : G \to \mathcal{U}(\mathfrak{u}(m))$ corresponds to the real unitary representation of G where it acts via conjugation on $\mathfrak{u}(m)$, and that ϕ corresponds to either ψ or its complexification, $\psi_{\mathbb{C}} : G \to \mathcal{U}(\mathfrak{gl}(m))$. In addition, suppose $i\mathbf{O} \in \mathfrak{u}(m)$ and V_1 and V_2 are irreducible representations satisfying the hypotheses of Supplementary Lemma 6, and $i\mathbf{O}_1, i\mathbf{O}_2$ orthogonal projections of $\mathbf{O}$ onto V_1 and V_2, respectively. Then*

$$\int_G \phi^{(1)}(g) i\mathbf{O}_1 \otimes \phi^{(2)}(g) i\mathbf{O}_2 dg = \frac{-\mathrm{Tr}(\mathbf{O}_1 \gamma \mathbf{O}_2)\tilde{\mathbf{K}}_{(1,2)}}{\dim V_1}, \tag{144}$$

where

$$\tilde{\mathbf{K}}_{(1,2)} := \sum_{j,k} u_j \otimes v_k \langle u_j, (\gamma^{-1})^\dagger v_k \rangle, \tag{145}$$

for two arbitrary orthonormal bases $\{u_j\}$ and $\{v_k\}$ for V_1 and V_2 respectively.

Proof. The orthogonal projections of $\mathbf{O}$ can be expressed in the bases defined earlier for V_1 and V_2 in the previous lemma: $i\mathbf{O}_1 + i\mathbf{O}_2 = \sum_j a_j u_j + \sum_k b_k v_j$. Let $n = \dim V_1 = \dim V_2$. Thus,

$$\begin{aligned}
n\int_G \phi^{(1)}(g)\mathbf{O}_1 \otimes \phi^{(2)}(g)\mathbf{O}_2 dg =& n\int_G \sum_j a_j \phi^{(1)}(g) u_j \otimes \sum_k b_k \phi^{(2)}(g) v_k dg & (146)\\
&= n\int_G \sum_j a_j \sum_{j'} \phi^{(1)}_{j',j}(g) u_{j'} \otimes \sum_k b_k \sum_{k'} \phi^{(2)}_{k',k}(g) v_{k'} dg & (147)\\
&= n \sum_{j,k,j',k'} a_j b_k \int_G \phi^{(1)}_{j',j}(g)\phi^{(2)}_{k',k}(g) dg \cdot u_{j'} \otimes v_{k'} & (148)\\
&= n \sum_{j,k,j',k'} a_j b_k \frac{\langle \gamma v_k, u_j\rangle \langle \gamma^{-1} u_{j'}, v_{k'}\rangle}{n} \cdot u_{j'} \otimes v_{k'} & (149)\\
&= \left(\sum_{j,k} a_j b_k \langle u_j, \gamma v_k\rangle\right)\left(\sum_{j,k} u_j \otimes v_k \langle u_j, (\gamma^{-1})^\dagger v_k\rangle\right) & (150)\\
&= -\mathrm{Tr}(\mathbf{O}_1\gamma\mathbf{O}_2)\left(\sum_{j,k} u_j \otimes v_k \langle u_j, (\gamma^{-1})^\dagger v_k\rangle\right) & (151)\\
&= -\mathrm{Tr}(\mathbf{O}_1\gamma\mathbf{O}_2)\tilde{\mathbf{K}}_{(1,2)}. & (152)
\end{aligned}$$

Note that $\tilde{\mathbf{K}}_{(1,2)}$ must be invariant under the choice of orthonormal bases for V_1 and V_2 since the left-hand side and $-\mathrm{Tr}(\mathbf{O}_1\gamma\mathbf{O}_2)$ are.

□

The next lemma presents expression for the covariance terms and the variance of the ideal complement.

Supplementary Lemma 8. *Suppose G is a compact matrix Lie group with Lie algebra $\mathfrak{g} \subseteq \mathfrak{u}(m)$, where $\mathfrak{u}(m)$ is the Lie algebra of $m \times m$ skew-Hermitian matrices. Suppose $\psi : G \to \mathcal{U}(\mathfrak{u}(m))$ corresponds to the real unitary representation of G where it acts via conjugation on $\mathfrak{u}(m)$, and that ϕ corresponds to either ψ or its complexification, $\psi_{\mathbb{C}} : G \to \mathcal{U}(\mathfrak{gl}(m))$. In addition, suppose $i\mathbf{O} \in \mathfrak{u}(m)$ and V_1 and V_2 are irreducible representations satisfying the hypotheses of Supplementary Lemma 6, and $i\mathbf{O}_1, i\mathbf{O}_2$ orthogonal projections of $\mathbf{O}$ onto V_1 and V_2, respectively. Lastly, $\forall g \in G, \mathbf{U}_g = \phi(g)$ and $\mathbf{H}$ is arbitrary element of $d\phi(\mathfrak{g})$. Then the following two equalities hold:*

$$\iint_G \mathrm{Tr}(\mathbf{U}_{g^-} i\rho \mathbf{U}_{g^-}^\dagger [\mathbf{H}, \mathbf{U}_{g^+} i\mathbf{O}_1 \mathbf{U}_{g^+}^\dagger])\mathrm{Tr}(\mathbf{U}_{g^-} i\rho \mathbf{U}_{g^-}^\dagger [\mathbf{H}, \mathbf{U}_{g^+} i\mathbf{O}_2 \mathbf{U}_{g^+}^\dagger]) dg^+ dg^- = \frac{(\mathbf{O},\rho)_{(1,2)}(i\mathbf{H})_{(1,2)}}{(\dim V_1)^2} \tag{153}$$

$$\iint_G (\mathrm{Tr}(\mathbf{U}_{g^-} i\rho \mathbf{U}_{g^-}^\dagger [\mathbf{H}, \mathbf{U}_{g^+} i\mathbf{O}_1 \mathbf{U}_{g^+}^\dagger]))^2 dg^+ dg^- = \frac{\|\mathbf{O}_1\|_{\mathrm{F}}^2 \|\rho_1\|_{\mathrm{F}}^2 (i\mathbf{H})_{(1,1)}}{(\dim V_1)^2}, \tag{154}$$

where $(\mathbf{O},\rho)_{1,2}$ and $(i\mathbf{H})_{(1,\cdot)}$ utilize Supplementary Definitions 3 and 4 respectively.

Proof. The first quantity in the emma statement, without taking the trace, is the following integral:

$$\text{Moment}_{(1,2)} := \iint_G \mathbf{U}_{g^-} i\rho \mathbf{U}_{g^-}^\dagger [\mathbf{H}, \mathbf{U}_{g^+} i\mathbf{O}_1 \mathbf{U}_{g^+}^\dagger] \otimes \mathbf{U}_{g^-} i\rho \mathbf{U}_{g^-}^\dagger [\mathbf{H}, \mathbf{U}_{g^+} i\mathbf{O}_2 \mathbf{U}_{g^+}^\dagger] dg^+ dg^-. \tag{155}$$

We can expand the commutator as in the proof of Theorem 2.8 of the main text to isolate out an integral of the form:

$$\iint_G \mathbf{U}_{g^+} i\mathbf{O}_1 \mathbf{U}_{g^+}^\dagger \otimes \mathbf{U}_{g^+} i\mathbf{O}_2 \mathbf{U}_{g^+}^\dagger dg^+ = \frac{-\text{Tr}(\mathbf{O}_1 \gamma \mathbf{O}_2) \tilde{\mathbf{K}}_{(1,2)}}{\dim W_1}, \tag{156}$$

where we have used Supplementary Lemma 7. If we fix arbitrary orthonormal bases for W_1 and W_2, $\{u_j\}$ and $\{v_j\}$ respectively, we can express $\tilde{\mathbf{K}}_{(1,2)}$ as

$$\tilde{\mathbf{K}}_{(1,2)} := \sum_{j,k} u_j \otimes v_k \langle u_j, (\gamma^{-1})^\dagger v_k \rangle. \tag{157}$$

If we plug Supplementary Equation (156) back into Supplementary Equation (155) as done in the proof of Theorem 2.8, then we get:

$$\begin{aligned}
\text{Moment}_{(1,2)} &= \frac{-\text{Tr}(\mathbf{O}_1 \gamma \mathbf{O}_2)}{\dim W_1} \sum_{j,k} \langle u_j, (\gamma^{-1})^\dagger v_k \rangle \int_G \mathbf{U}_{g^-} [\mathbf{H}, u_j] \mathbf{U}_{g^-}^\dagger \otimes \mathbf{U}_{g^-} [\mathbf{H}, v_k] \mathbf{U}_{g^-}^\dagger dg^- && (158)\\
&= \frac{-\text{Tr}(\mathbf{O}_1 \gamma \mathbf{O}_2)}{(\dim W_1)^2} \left(\sum_{j,k} -\langle u_j, (\gamma^{-1})^\dagger v_k \rangle \text{Tr}(i[\mathbf{H}, u_j] \gamma i [\mathbf{H}, v_k]) \right) \left(\sum_{j,k} u_j \otimes v_k \langle u_j, (\gamma^{-1})^\dagger v_k \rangle \right) && (159)\\
&= \frac{-\text{Tr}(\mathbf{O}_1 \gamma \mathbf{O}_2)(i\mathbf{H})_{(1,2)}}{(\dim W_1)^2} \left(\sum_{j,k} u_j \otimes v_k \langle u_j, (\gamma^{-1})^\dagger v_k \rangle \right) && (160)
\end{aligned}$$

where we have applied Supplementary Lemma 7 again to obtain the second equality. Also, we have utilized Supplementary Definition 4 and that $[\mathbf{H}, \cdot]$ preserves W_1 and W_2.

Finally, the overall integral is

$$\begin{aligned}
\text{Tr}(i\rho \otimes i\rho \cdot \text{Moment}_{(1,2)}) &= \frac{-\text{Tr}(\mathbf{O}_1 \gamma \mathbf{O}_2)(i\mathbf{H})_{(1,2)}}{(\dim W_1)^2} \left(\sum_{j,k} \text{Tr}(i\rho u_j) \text{Tr}(i\rho v_k) \langle u_j, (\gamma^{-1})^\dagger v_k \rangle \right) && (161)\\
&= \frac{\text{Tr}(\mathbf{O}_1 \gamma \mathbf{O}_2) \text{Tr}(\rho (\gamma^{-1})^\dagger \rho)(i\mathbf{H})_{(1,2)}}{(\dim W_1)^2} && (162)\\
&= \frac{(\mathbf{O}, \rho)_{(1,2)} (i\mathbf{H})_{(1,2)}}{(\dim W_1)^2}. && (163)
\end{aligned}$$

The second equality in the lemma follows by notating that γ becomes the identity map, and u_j, v_k are both indexing elements of the same orthonormal basis for V_1. The existence of G-isomorphic V_2 is not needed.

□

Now we have all the tools to prove Supplementary Theorem 5.

Proof of Supplementary Theorem 5. Recall that

$$\begin{aligned}
\text{GradVar} \geq & \text{Var}_{(g_+, g_-) \sim \mu_\alpha^{\otimes 2}} [\partial \langle \mathbf{O}_\mathfrak{g} \rangle_\rho] + \text{Var}_{(g_+, g_-) \sim \mu^{\otimes 2}} [\partial \langle \mathbf{O}_{\mathfrak{g}^c_{\cong}} \rangle_\rho] + \\
& + 2\text{Cov}_{(g_+, g_-) \sim \mu^{\otimes 2}} [\partial \langle \mathbf{O}_\mathfrak{g} \rangle_\rho, \partial \langle \mathbf{O}_{\mathfrak{g}^c_{\cong}} \rangle_\rho]. && (164)
\end{aligned}$$

We already know the expressions for $\text{Var}_{(g_+, g_-) \sim \mu_\alpha^{\otimes 2}} [\partial \langle \mathbf{O}_\mathfrak{g} \rangle]$, which follows from Theorem 2.9 of the main text. There are two kinds of terms that will be in $\text{Var}_{(g_+, g_-) \sim \mu^{\otimes 2}} [\partial \langle \mathbf{O}_{\mathfrak{g}^c_{\cong}} \rangle]$. The first kind are just variance terms from the ideal-complement and the second kind are covariances between terms in the ideal-complement that are isomorphic to each other. We can deal with the second class of terms in a similar way to how we deal with $2\text{Cov}_{(g_+, g_-) \sim \mu^{\otimes 2}} [\partial \langle \mathbf{O}_\mathfrak{g} \rangle, \partial \langle \mathbf{O}_{\mathfrak{g}^c_{\cong}} \rangle]$.

Note that since we assumed $\mathfrak{g}$ is a compact Lie algebra, it follows that the second condition of Supplementary Supplementary Lemma 6 is satisfied. This is because any irreducible representation isomorphic to a simple ideal of

compact real Lie algebra remains irreducible when complexified. Thus all of the previous lemmas apply for the ideal and ideal-complement components.

Thus using Theorem 2.9 of the main text and Supplementary Lemma 8 we get that:

$$\mathrm{Var}_{(g_+,g_-)\sim\mu_\alpha^{\otimes 2}}[\partial\langle \mathbf{O}_{\mathfrak{g}}\rangle] = \sum_\alpha \frac{\|\mathbf{H}_{\mathfrak{g}_\alpha}\|_{\mathrm{K}}^2 \|\mathbf{O}_{\mathfrak{g}_\alpha}\|_{\mathrm{F}}^2 \|\rho_{\mathfrak{g}_\alpha}\|_{\mathrm{F}}^2}{d_{\mathfrak{g}_\alpha}^2} \tag{165}$$

$$\mathrm{Var}_{(g_+,g_-)\sim\mu^{\otimes 2}}[\partial\langle \mathbf{O}_{\mathfrak{g}^c_{\cong}}\rangle] + 2\mathrm{Cov}_{(g_+,g_-)\sim\mu^{\otimes 2}}[\partial\langle \mathbf{O}_{\mathfrak{g}}\rangle, \partial\langle \mathbf{O}_{\mathfrak{g}^c_{\cong}}\rangle]$$
$$= \sum_{\kappa|\kappa\cong\alpha} (\|\mathbf{O}_\kappa\|_{\mathrm{F}}^2 \|\rho_\kappa\|_{\mathrm{F}}^2 (i\mathbf{H})_{(\kappa,\kappa)} + \sum_{\{\kappa'|\kappa'\cong\kappa\}\cup\{\alpha\}} (\mathbf{O},\rho)_{(\kappa,\kappa')}(i\mathbf{H})_{(\kappa,\kappa')})). \tag{166}$$

□

B. Variance of Non-ideal Complement

One may have noticed that the lemmas derived previously were actually general enough that they can allow us to exactly compute the variance of the non-ideal complement.

The non-ideal complement component of the variance corresponds to those irreducible components W_κ that are not G-isomorphic to any simple ideal of $\mathfrak{g}$. In this case, it is not necessarily true that complexification of W_κ remains irreducible. However, if it is not irreducible, it is known that it must decompose into exactly two complex irreducible representations of G [14]. This allows us to then apply the techniques of Supplementary Note 6 to these complex irreps.

We start by complexifying the representation ψ, which is done by linearly extending ψ to the complex vector space $\mathfrak{u}(V) + i\mathfrak{u}(V) \cong \mathfrak{gl}(V)$. The extended inner product is defined by: $\mathbf{A}_1 + i\mathbf{A}_2, \mathbf{B}_1 + i\mathbf{B}_2 \in \mathfrak{u}(V) + i\mathfrak{u}(V)$: $-\mathrm{Tr}((\mathbf{A}_1 + i\mathbf{A}_2)(\mathbf{B}_1 - i\mathbf{B}_2))$. In addition, the complexified representation remains unitary w.r.t. this inner product. Thus, $\mathfrak{gl}(V)$ breaks into the following sum of orthogonal components:

$$\mathfrak{gl}(V) = \bigoplus_\alpha (d\phi(\mathfrak{g}_\alpha) + i d\phi(\mathfrak{g}_\alpha)) \oplus \left(\bigoplus_\alpha \bigoplus_{\kappa\cong\alpha} (W_\kappa + iW_\kappa)\right) \oplus \left(\bigoplus_{\forall\alpha,\kappa\ncong\alpha} (W_\kappa + iW_\kappa)\right). \tag{167}$$

The first two sums are the ideal and ideal complement components and remain irreducible when complexified. These were handled in the Supplementary Note 6. However, as stated in the previous paragraph, the terms in the last sum, which correspond to the non-ideal complement, will either remain irreducible or split into two complex irreducible components. Thus, we can further break the sum up as follows:

$$\mathfrak{gl}(V) = \bigoplus_\alpha (d\phi(\mathfrak{g}_\alpha) + i d\phi(\mathfrak{g}_\alpha)) \oplus \left(\bigoplus_\alpha \bigoplus_{\kappa\cong\alpha} (W_\kappa + iW_\kappa)\right) \oplus \left(\bigoplus_{\forall\alpha,\kappa\ncong\alpha\&\mathbb{C}-\mathrm{irrep}} (W_\kappa + iW_\kappa)\right) \tag{168}$$

$$\oplus \left(\bigoplus_{\forall\alpha,\kappa\ncong\alpha\&\mathrm{not}\mathbb{C}-\mathrm{irrep}} (V_\kappa + V_\kappa^*)\right), \tag{169}$$

where the V_κ are complex irreps and * denotes the dual representation, which it may or may not be isomorphic to V_κ. Schur orthogonality now applies, and the covariances terms across an $\oplus$ will be annihilated, and since each V_κ is complex irreducible all of the results of Schur's lemma apply.

Thus, using that our observable $\mathbf{O}$ satisfies $i\mathbf{O} \in \mathfrak{u}(V)$ the non-ideal variance component will consist of terms of the form:

$$\frac{1}{\dim \tilde{W}_\kappa} \left(\|\mathbf{O}_\kappa\|_{\mathrm{F}}^2 \|\rho_\kappa\|_{\mathrm{F}}^2 (i\mathbf{H})_{(\kappa,\kappa)} + \sum_{\kappa'|\kappa'\cong\kappa} (\mathbf{O},\rho)_{(\kappa,\kappa')}(i\mathbf{H})_{(\kappa,\kappa')} \right). \tag{170}$$

where $\tilde{W}_\kappa$ may correspond to either a real or complex irrep. Since $\tilde{W}_\kappa$ can be a complex vector space, the variance scaling can depend on the dimension of a vector space that contains operators that are not valid quantum observables, i.e. the spaces W_κ, and a novel prediction of our work. This emphasizes that the variance of the non-ideal complement

can have no dependence on the dimension of the DLA, yet still, the techniques we utilizes for the LASA setting still work in general for decomposing the variance and identifying the invariant-subspace dimension dependence.

Thus, if $\mathbf{O}$ only has support on the non-ideal complement, all scenarios are possible. If $\mathfrak{g}$ has polynomially growing dimension, then the complement is exponential, and potentially there exists an exponentially-large irreducible subspace that would give a BP. Conversely, if $\mathfrak{g}$ is exponentially large and its complement is polynomial, it may be that some choices of $\mathbf{O}$ and ρ would avoid a BP.

Supplementary Note 7 – Details of Numerical Results

The numerical results displayed in the figures in the main text were obtained with Qulacs. For each simulation a periodic ansatz using the quantum compound generators was constructed. Specifically, the ansatz is in the brick architecture as described in Ref. [15], with alternating layers of 2-qubit gates with 1D connectivity. Since the experiments focused on the SU-compound, each gate was composed of a regular FBS gate (generator h_y) followed by a generalized FBS gate (generated h_x), independently parameterized, in order to express the entire group.

The experiments used $L = 12n$ layers, where n is the number of qubits. The parameters were uniformly sampled from $[0, 4\pi)$. The scaling of L appeared to be sufficient for constructing an approximate 2-design. The gradients were estimated via finite difference, and the variance was estimated over 5000 gradient samples for $n < 18$ and 1000 for $n \geq 18$, due to time constraints.

Due to a different convention for the Pauli generators in the simulation software used, we rescaled the analytical predictions in the plots by a factor of 1/4.

Supplementary Note 8 – Mixing time to t-designs

In this section, we prove Theorem 2.6 of the main text regarding the mixing time to 2-design for LASA. However, the result proved below is slightly more general and Theorem 2.6 follows when we restrict the DLA to have polynomial dimension and a 2-design.

First, we start with a definition. Let, $\{\mathbf{H}_1, \ldots, \mathbf{H}_{d_\mathfrak{g}}\}$ be a basis of skew-Hermitian operators for the Lie algebra $\mathfrak{g}$. We define the *minimum stable Killing rank* r_K to be

$$r_\mathrm{K} = \min_k \frac{\|\mathrm{ad}_{i\mathbf{H}_k}\|_\mathrm{F}^2}{\|\mathrm{ad}_{i\mathbf{H}_k}\|_\mathrm{op}^2}. \tag{171}$$

Note that $1 \leq r_\mathrm{K} \leq d_\mathfrak{g}$. However, we can actually show that $r_\mathrm{K} \in \Omega(\sqrt{d_\mathfrak{g}})$ by investigating the potential root systems (see Lemma 9). Corollary 6.2 shows that the quantum compound ansatz saturates this lower bound. In contrast Ref. [16] showed that Pauli rotations for $\mathfrak{su}(2^n)$ saturate the upper bound.

Supplementary Theorem 6. *Consider an orthogonal basis of skew-Hermitian generators $\mathcal{A} := \{\mathbf{H}_1, \ldots, \mathbf{H}_{d_\mathfrak{g}}\}$ for the DLA with the property that the unitary $e^{-\theta\mathbf{H}_k}$ corresponding to a generator $\mathbf{H}_k$ is t_k-periodic. Suppose $\mathbf{H}_k$ constitute an irreducible representation of the dynamical group $\mathcal{G}$. Consider a LASA formed by applying evolutions $e^{-\theta_k\mathbf{H}_k}$ where $\mathbf{H}_k$ is selected uniformly at random from the set $\mathcal{A}$ and the parameter θ_k uniformly from $[0, t_k)$. Then, when $t < \sqrt{d_g}/2$, the ansatz is an ϵ-approximate t-design for the dynamical group $\mathcal{G}$ after $\mathcal{O}(\frac{td_\mathfrak{g}}{r_k}\log(1/\epsilon))$ layers.*

Proof. The proof roughly follows the approach of Ref. [16] with some generalizations made to handle arbitrary LASA.

Suppose the dynamical group is $\mathcal{G}$ and is an irreducible representation, ϕ, of some compact, connected group G. The irreducibility assumption can be satisfied by restricting to the projection of the initial state onto an irreducible invariant subspace of the n-qubit Hilbert space.

We can without loss of generality assume that the Lie algebra $\mathfrak{g}$ is also simple. This is because mixing on each simple ideal will correspond to mixing over the whole group. Then, we can consider the minimal spectral gap across all ideals.

Consider the random walk that at each step uniformly selects an element from $\{\mathbf{H}_k\}_{k=1}^{d_\mathfrak{g}}$ and an angle from $\theta \in [0, t_k]$ and applies $e^{-\theta\mathbf{H}_k}$. The t-th moment corresponding to the walk is:

$$T := \sum_{k=1}^{d_\mathfrak{g}} \frac{1}{d_\mathfrak{g}} \int_{[0,t_k]} (e^{-\theta_k\mathbf{H}_k})^{\otimes t} \otimes (e^{\theta_k\bar{\mathbf{H}}_k})^{\otimes t} d\theta_k, \tag{172}$$

and let

$$T^* := \int_G (\mathbf{U}_g)^{\otimes t} \otimes (\bar{\mathbf{U}}_g)^{\otimes t} dg \tag{173}$$

be the t-th Haar moment. The spectral gap [17, 18] of the walk is

$$\Delta := 1 - \|T - T^*\|_{\text{op}}, \tag{174}$$

where $\|\cdot\|_{\text{op}}$ denotes the operator norm. The gap quantifies the complexity of forming an approximate t-design as we can exponentially suppress the error with additional steps.

The integral

$$\int_{[0,t_k]} (e^{-\theta_k \mathbf{H}_k})^{\otimes t} \otimes (e^{\theta_k \bar{\mathbf{H}}_k})^{\otimes t} d\theta_k \tag{175}$$

is an orthogonal projection onto commutant of $\{(e^{-\theta_k \mathbf{H}_k})^{\otimes t}, \theta_k \in [0, t_k]\}$ or equivalently the kernel of

$$\sum_{r=1}^{t} \mathbb{1}^{\otimes(r-1)} \otimes (-\mathbf{H}_k \otimes \mathbb{1} + \mathbb{1} \otimes \bar{\mathbf{H}}) \otimes \mathbb{1}^{\otimes(t-r)}. \tag{176}$$

We will denote the orthogonal projector onto the kernel of $\mathbf{A}$ by $\text{Ker}(\mathbf{A})$. In addition due the assumption of LASA, $(-\mathbf{H}_k \otimes \mathbb{1} + \mathbb{1} \otimes \bar{\mathbf{H}})$ only acts on tensors corresponding to vectorized forms of skew-Hermitian matrices, and we can thus ignore the trivial representation component. Under this assumption, we can equivalently consider the kernel of

$$\text{ad}_{\mathbf{H}_k}^{\otimes t} := \sum_{r=1}^{t} \mathbb{1}^{\otimes(r-1)} \otimes \text{ad}_{\mathbf{H}_\text{k}} \otimes \mathbb{1}^{\otimes(t-r)}, \tag{177}$$

which corresponds to the t-th tensor power of the adjoint.

Since the representation $\text{ad}^{\otimes t}$ is completely reducible, and thus decomposes into irreps ψ_m, the integral must respect this decomposition:

$$\int_{[0,t_k]} (e^{-\theta_k \mathbf{H}_k})^{\otimes 2} \otimes (e^{-\theta_k \mathbf{H}_k^*})^{\otimes 2} d\theta_k = \text{Ker}(\text{ad}_{\mathbf{H}_k}^{\otimes t}) \tag{178}$$

$$= \bigoplus_m \text{Ker}(\psi_m(\mathbf{H}_k)). \tag{179}$$

If we consider the max over all m that don't correspond to the trivial rep, since T^* and T agree on the trivial components, we have

$$\|T - T^*\|_{\text{op}} = \|\frac{1}{d_{\mathfrak{g}}} \sum_{k=1}^{d_{\mathfrak{g}}} \bigoplus_m \text{Ker}(\psi_m(\mathbf{H}_k))\|_{\text{op}} \tag{180}$$

$$\leq \max_m \frac{1}{d_{\mathfrak{g}}} \|\sum_{k=1}^{d_{\mathfrak{g}}} \text{Ker}(\psi_m(\mathbf{H}_k))\|_{\text{op}}. \tag{181}$$

Note that the first inequality follows because including more projectors only increases the operator norm. One can verify that

$$\text{Ker}(\psi_m(\mathbf{H}_k)) \prec \mathbb{1} - \frac{\psi_m(\mathbf{H}_k)^2}{\|\psi_m(\mathbf{H}_k)\|_{\text{op}}^2}, \tag{182}$$

which gives:

$$d_{\mathfrak{g}}\mathbb{1} - \sum_{k=1}^{d_{\mathfrak{g}}} \mathrm{Ker}(\psi_m(\mathbf{H}_k)) \succ \sum_{k=1}^{d_{\mathfrak{g}}} \frac{\psi_m(\mathbf{H}_k)^2}{\|\psi_m(\mathbf{H}_k)\|_{\mathrm{op}}^2} \tag{183}$$

$$= \sum_{k=1}^{d_{\mathfrak{g}}} \frac{\|\mathbf{H}_k\|_{\mathrm{K}}^2}{\|\psi_m(\mathbf{H}_k)\|_{\mathrm{op}}^2} \frac{\psi_m(\mathbf{H}_k)^2}{\|\mathbf{H}_k\|_{\mathrm{K}}^2} \tag{184}$$

$$\succ \left(\min_k \frac{\|\mathbf{H}_k\|_{\mathrm{K}}^2}{\|\psi_m(i\mathbf{H}_k)\|_{\mathrm{op}}^2}\right) \sum_{k=1}^{d_{\mathfrak{g}}} \frac{-\psi_m(\mathbf{H}_k)^2}{\|\mathbf{H}_k\|_{\mathrm{K}}^2} \tag{185}$$

$$= \left(\min_k \frac{\|\mathbf{H}_k\|_{\mathrm{K}}^2}{\|\psi_m(i\mathbf{H}_k)\|_{\mathrm{op}}^2}\right) C_{\psi_m} \tag{186}$$

$$\succ \left(\min_k \frac{\|\mathbf{H}_k\|_{\mathrm{K}}^2}{\|\psi_m(i\mathbf{H}_k)\|_{\mathrm{op}}^2}\right) c_{\psi_m}\mathbb{1} \tag{187}$$

$$\tag{188}$$

where C_{ψ_m} is the quadratic Casimir in the representation ψ_m. Due to $\mathfrak{g}$ being simple and ϕ irreducible, the $\psi_m(\mathbf{H}_k)$ are orthogonal if the $\mathbf{H}_k$ are. Since ψ_m is irreducible Schur's lemma gives that $C_{\psi_m} = c_{\psi_m}\mathbb{1}$. Thus,

$$\Delta := 1 - \|T - T^*\|_{\mathrm{op}} \tag{189}$$

$$\geq 1 - \max_m \frac{1}{d_{\mathfrak{g}}} \|\sum_{k=1}^{d_{\mathfrak{g}}} \mathrm{Ker}(\psi_m(i\mathbf{H}_k))\|_{\mathrm{op}} \tag{190}$$

$$= \min_m \frac{1}{d_{\mathfrak{g}}} \|d_{\mathfrak{g}}\mathbb{1} - \sum_{k=1}^{d_{\mathfrak{g}}} \mathrm{Ker}(\psi_m(\mathbf{H}_k))\|_{\mathrm{op}} \tag{191}$$

$$= \min_{m|m \text{ non-trivial}} \left(\min_k \frac{\|\mathbf{H}_k\|_{\mathrm{K}}^2}{\|\psi_m(i\mathbf{H}_k)\|_{\mathrm{op}}^2}\right) \frac{c_{\psi_m}}{d_{\mathfrak{g}}} \tag{192}$$

$$\geq \min_{m|m \text{ non-trivial}} \left(\min_k \frac{\|\mathbf{H}_k\|_{\mathrm{K}}^2}{t^2\|\mathrm{ad}_{i\mathbf{H}_k}\|_{\mathrm{op}}^2}\right) \frac{c_{\psi_m}}{d_{\mathfrak{g}}}. \tag{193}$$

Since $\mathfrak{g}$ is compact simple, it must be isomorphic to either $\mathfrak{su}(m)$, $\mathfrak{so}(m)$, or $\mathfrak{sp}(m)$, and corresponding comlexified algebras are $\mathfrak{sl}(\mathbb{C})$, $\mathfrak{so}(\mathbb{C})$ or $\mathfrak{sp}(\mathbb{C})$. For analyzing the eigenvalue of the Casimir we need to look at the root system for the complexified algebra.

For a highest weight λ, the eigenvalue of the Casimir is

$$\langle\lambda,\lambda\rangle + \langle\lambda, 2\delta\rangle, \tag{194}$$

where δ is the Weyl vector for the given root system. In addition, the inner product is w.r.t. the standard Euclidean inner product, and thus to account for our chosen normalization of the $\mathbf{H}_k$ w.r.t. the Killing form, we need to divide by $I_{\mathrm{Ad}} \in \Theta(\sqrt{d_{\mathfrak{g}}})$. So really we have:

$$c_{\psi_m} \in \Theta\left(\frac{\langle\lambda_{\psi_m},\lambda_{\psi_m}\rangle + \langle\lambda_{\psi_m}, 2\delta\rangle}{\sqrt{d_{\mathfrak{g}}}}\right), \tag{195}$$

for irrep ψ_m. Note that below $n \in \Theta(\sqrt{d_{\mathfrak{g}}})$.

Following Ref. [2], the four possible root systems $A_n = \mathfrak{sl}(n+1)$, $B_n = \mathfrak{so}(2n,\mathbb{C})$, $C_n = \mathfrak{sp}(2n,\mathbb{C})$, and $D_n = \mathfrak{so}(2n+1,\mathbb{C})$ are:

$$A_n : e_j - e_k, j \neq k \tag{196}$$

$$B_n : \pm e_j \pm e_k, j \neq k \cup \pm e_j \tag{197}$$

$$C_n : \pm e_j \pm e_k, j \neq k \cup \pm 2e_j \tag{198}$$

$$D_n : \pm e_j \pm e_k, j \neq k. \tag{199}$$

Each has as a basis:

$$A_n : e_j - e_{j+1}, j = 1, \ldots, n \tag{200}$$
$$B_n : e_j - e_{j+1}, j = 1, \ldots, n-1 \cup e_n \tag{201}$$
$$C_n : e_j - e_{j+1}, j = 1, \ldots, n-1 \cup 2e_n \tag{202}$$
$$D_n : e_j - e_{j+1}, j = 1, \ldots, n-1 \cup e_{n-1} + e_n, \tag{203}$$

where the positive roots can be expressed as positive linear combinations of the above basis elements. Thus the positive roots for each system are:

$$A_n : \{e_j - e_k \mid j, k \in \{1, \ldots, n+1\} \ \& \ j < k\} \tag{204}$$
$$B_n : \{e_j \pm e_k, e_j \mid j, k \in \{1, \ldots, n\} \ \& \ j < k\} \tag{205}$$
$$C_n : \{e_j \pm e_k, 2e_j \mid j, k \in \{1, \ldots, n\} \ \& \ j < k\} \tag{206}$$
$$D_n : \{e_j \pm e_k \mid j, k \in \{1, \ldots, n\} \ \& \ j < k\}. \tag{207}$$

The Weyl vector is one-half the sum of the positive roots leading to

$$2\delta_j^{(A)} = n - 2(j-1) \tag{208}$$
$$2\delta_j^{(B)} = 2n - 2j + 1 \tag{209}$$
$$2\delta_j^{(C)} = 2n - 2j + 2 \tag{210}$$
$$2\delta_j^{(D)} = 2n - 2j. \tag{211}$$

A necessary condition a highest weight λ that is common across all four systems is that the inner product between λ and each base root (coroot) must be ≥ 0 and integer. In other words, it is a dominant integral element of the lattice. We now look at the possible highest weight vectors of the reducible representation $\mathrm{ad}^{\otimes t}$ for each of the root systems to lower bound the eigenvalue of the Casimir.

For A_n such vectors λ in $\mathbb{R}^{n+1}$ in basis $\{e_j\}_{j=1}^{n+1}$ must satisfy

$$\lambda_j \in \mathbb{Z} \tag{212}$$
$$\lambda_j \geq \lambda_{j+1} \tag{213}$$
$$\sum_j |\lambda_j| = 2t, \tag{214}$$

and be a linear combination of t roots. Thus one can verify that the minimizer of the Casimir eigenvalue under such constraints is $\sum_{j=1}^t e_j - e_{n+2-j}$. This leads to

$$c_{\psi_m} \in \Omega\left(\frac{2t(n-t+2)}{n}\right). \tag{215}$$

The reproduces the result of Ref.[16].

For B_n in the basis $\{e_j\}_{j=1}^n$ we have that a highest weight in $\mathbb{R}^n$ must satisfy

$$\lambda_j \in \mathbb{Z} \tag{216}$$
$$\lambda_j \geq \lambda_{j+1} \geq 0 \tag{217}$$
$$\sum_j |\lambda_j| \leq 2t, \tag{218}$$

and be a linear combination of t roots. This gives a minimizer of $\sum_{j=1}^t e_j$, leading to

$$c_{\psi_m} \in \Omega\left(\frac{t(2n-t+2)}{n}\right), \tag{219}$$

which also applies for C_n.

Lastly, for D_n we have the following constraints for a highest weight in $\mathbb{R}^n$ we again have

$$\lambda_j \in \mathbb{Z} \tag{220}$$
$$\lambda_j \geq \lambda_{j+1} \geq 0 \tag{221}$$
$$\sum_j |\lambda_j| \leq 2t, \tag{222}$$

and be a linear combination of t roots. This leads to a minimizer of $\sum_{j=1}^t (e_j + e_{j+1})$. However, we can actually just use $\sum_{j=1}^t e_j$ as a lower bound leading to

$$c_{\psi_m} \in \Omega\left(\frac{t(2n-t)}{n}\right). \tag{223}$$

The conclusion is that in all cases, we see that for $t \leq n$:

$$\frac{c_{\psi_m}}{t^2} \in \Omega(1/t), \tag{224}$$

so we can conclude that

$$\Delta \in \Omega\left(\min_k \frac{\|\mathbf{H}_k\|_{\mathrm{K}}^2}{\|\mathrm{ad}_{i\mathbf{H}_k}\|_{\mathrm{op}}^2 d_{\mathfrak{g}} t}\right) = \Omega\left(\min_k \frac{\|\mathrm{ad}_{i\mathbf{H}_k}\|_{\mathrm{F}}^2}{\|\mathrm{ad}_{i\mathbf{H}_k}\|_{\mathrm{op}}^2 d_{\mathfrak{g}} t}\right) = \Omega\left(\frac{r_{\mathrm{K}}}{d_{\mathfrak{g}} t}\right), \tag{225}$$

where $r_{\mathrm{K}} := \min_k \frac{\|\mathrm{ad}_{i\mathbf{H}_k}\|_{\mathrm{F}}^2}{\|\mathrm{ad}_{i\mathbf{H}_k}\|_{\mathrm{op}}^2}$ is the minimum stable rank of the adjoint representation w.r.t. the chosen DLA basis. □

The spectral gap result of Ref. [16] for $\mathfrak{su}(2^n)$ and the basis of Pauli operators follows from noting that under these conditions $r_{\mathrm{K}} \in \Theta(d_{\mathfrak{g}})$. Thus the mixing can be efficient for exponential DLAs.

Supplementary Lemma 9. *For any compact simple Lie algebra, $r_K \in \Omega(\sqrt{d_{\mathfrak{g}}})$.*

Proof. The value of r_{K} only depends on elements from the Cartan subalgebra. In addition, for compact simple Lie algebra, all elements are conjugate to an element from a Cartan subalgebra and all Cartan subalgebra are conjugate.

We can express elements from the Cartan subalgebra using a basis for the root systems (i.e. (204)–(207)). Specifically let $\{\alpha_k\}_{k=1}^n$ denote a basis for one of the four root systems A_n, B_n, C_n, or D_n. Let $\mathcal{R}$ denote the total set of roots, and for any two roots α, β, $|\langle \alpha, \beta \rangle| \leq 2$ (see (204)– (207)), where $\langle \cdot, \cdot \rangle$ is the standard Euclidean inner product on the root system. Note that

$$r_{\mathrm{K}}^{(j)} = \frac{\|\mathrm{ad}_{i\mathbf{H}_j}\|_{\mathrm{F}}^2}{\|\mathrm{ad}_{i\mathbf{H}_j}\|_{\mathrm{op}}} = \frac{\sum_{\alpha \in \mathcal{R}} \langle \alpha, i\mathbf{H}_j \rangle^2}{\max_{\alpha \in \mathcal{R}} \langle \alpha, i\mathbf{H}_j \rangle^2}. \tag{226}$$

We can express $\mathbf{H}_j$ in terms of the α_k, $i\mathbf{H}_j = \sum_{k=1}^n c_k \alpha_k$ Note that we slightly abuse notation since $\mathbf{H}_j$ here is really the preimage of $\mathbf{H}_j$ under the representation ϕ. However, the Lie algebra being simple implies that the adjoint representations are isomorphic if ϕ is not trivial. We also have

$$\sum_{\alpha \in \mathcal{R}} \langle \alpha_k, \alpha \rangle^2 \in \Omega(n) \in \Theta(\sqrt{d_{\mathfrak{g}}}), \tag{227}$$

as one can check using (204)-(207) and (200)–(203). Thus, we get

$$r_{\mathrm{K}}^{(j)} = \frac{\sum_{k=1}^n c_k^2 \sum_{\alpha \in \mathcal{R}} \langle \alpha_k, \alpha \rangle^2}{\max_{\alpha \in \mathcal{R}} \sum_{k=1}^n c_k^2 \langle \alpha_k, \alpha \rangle^2} \geq \frac{\sum_{k=1}^n c_k^2 \sum_{\alpha \in \mathcal{R}} \langle \alpha_k, \alpha \rangle^2}{\sum_{k=1}^n 4 c_k^2} \in \Omega(\sqrt{d_{\mathfrak{g}}}), \tag{228}$$

so $r_{\mathrm{K}} \in \Omega(\sqrt{d_{\mathfrak{g}}})$. □

The orthogonality assumption plays a key role in lower bounding the gap using the quadratic Casimir, and seems like an intuitive requirement for faster mixing. However, it is unclear if the full basis assumption can be relaxed and replaced with a dense subset. Numerical evidence appears to showing fast mixing when this condition is relaxed, for example QAOA-like ansatz [13]. Still, for sufficiently large m, the Baker-Campbell-Hausdorff formula gives that for any $\mathbf{H}_j, \mathbf{H}_k$:

$$\left(e^{-\sqrt{t/m}\mathbf{H}_j} e^{-\sqrt{t/m}\mathbf{H}_k} e^{\sqrt{t/m}\mathbf{H}_j} e^{\sqrt{t/m}\mathbf{H}_k}\right)^m = e^{-t[\mathbf{H}_j, \mathbf{H}_k] + \mathcal{O}(t^{3/2}/m^{1/2})}, \tag{229}$$

which can approximate nested commutator for large enough m. This is by no means showing that the full basis assumption can be relaxed for faster mixing. However, it shows that a subset of the generators can be used to approximately sample from their nested commutators, and the density of a periodic ansatz within its dynamical Lie group. The mixing of dense subgroups (i.e. nonzero spectral gap) seems to require further conditions [19, 20].

We now present two simple corollaries of Theorem 6 that appeared as theorems in the main text.

Supplementary Corollary 6.1 (Main Text Theorem 2.6)**.** *Consider an orthogonal basis of skew-Hermitian generators* $\mathcal{A} := \{\mathbf{H}_1, \ldots, \mathbf{H}_{d_\mathfrak{g}}\}$ *for the DLA with the property that the unitary* $e^{-\theta \mathbf{H}_k}$ *corresponding to a generator* $\mathbf{H}_k$ *is* t_k*-periodic. In addition, suppose that* $d_\mathfrak{g} = \mathcal{O}(\mathrm{poly}(n))$*. Consider a LASA formed by applying evolutions* $e^{-\theta_k \mathbf{H}_k}$ *where* $\mathbf{H}_k$ *is selected uniformly at random from the set* $\mathcal{A}$ *and the parameter* θ_k *uniformly from* $[0, t_k)$*. Then, the ansatz is an* ϵ*-approximate* 2*-design for the dynamical group* $\mathcal{G}$ *after* $\mathcal{O}(\mathrm{poly}(n)\log(1/\epsilon))$ *layers.*

Proof. One can see that the theorem follows from the lower bound on r_K. □

Supplementary Corollary 6.2 (Main Text Theorem 2.10)**.** *Consider an* n*-qubit quantum compound ansatz that is a LASA constructed using the set of generators* $\{X^{(ij)}, Y^{(ij)}, \sum_{i=1}^{j} Z^{(ij)}\}$ *with rotations angles chosen uniformly at random. Then, for* $t \leq n/2$*, the ansatz is an* ϵ*-approximate* t*-design for the dynamical group* $\mathrm{SU}(n)$ *after* $\mathcal{O}(tn\log(1/\epsilon))$ *layers.*

Proof. One can check that the chosen set of generators form an orthogonal basis for the DLA. Each of the $X^{(ij)}$ and $Y^{(ij)}$ Givens rotation generators is conjugate to an $Z^{(ij)}$ generator, which is an element of the Cartan subalgebra. Thus from Lemma 9 the result follows. □

---

[1] Anthony W Knapp and Anthony William Knapp, *Lie groups beyond an introduction*, Vol. 140 (Springer, 1996).
[2] Brian C Hall and Brian C Hall, *Lie groups, Lie algebras, and representations* (Springer, 2013).
[3] William Fulton and Joe Harris, *Representation theory: a first course*, Vol. 129 (Springer Science & Business Media, 2013).
[4] Roe Goodman, Nolan R Wallach, *et al.*, *Symmetry, representations, and invariants*, Vol. 255 (Springer, 2009).
[5] James E Humphreys, *Introduction to Lie algebras and representation theory*, Vol. 9 (Springer Science & Business Media, 2012).
[6] Terence Tao, *Hilbert's fifth problem and related topics*, Vol. 153 (American Mathematical Soc., 2014).
[7] Antonio Anna Mele, "Introduction to haar measure tools in quantum information: A beginner's tutorial," (2023), arXiv:2307.08956 [quant-ph].
[8] Jürgen Fuchs, *Affine Lie algebras and quantum groups: An Introduction, with applications in conformal field theory* (Cambridge university press, 1995).
[9] Howard E Haber, "The eigenvalues of the quadratic casimir operator and second-order indices of a simple lie algebra," `http://scipp.ucsc.edu/~haber/webpage/Casimir3.pdf`.
[10] Michael Ragone, Bojko N. Bakalov, Frédéric Sauvage, Alexander F. Kemper, Carlos Ortiz Marrero, Martin Larocca, and M. Cerezo, "A unified theory of barren plateaus for deep parametrized quantum circuits," (2023), arXiv:2309.09342 [quant-ph].
[11] Tobias Diez and Lukas Miaskiwskyi, "Expectation values of polynomials and moments on general compact lie groups," (2022), arXiv:2203.11607 [math.PR].
[12] Jürgen Fuchs and Christoph Schweigert, *Symmetries, Lie algebras and representations: A graduate course for physicists* (Cambridge University Press, 2003).
[13] Martin Larocca, Piotr Czarnik, Kunal Sharma, Gopikrishnan Muraleedharan, Patrick J. Coles, and M. Cerezo, "Diagnosing Barren Plateaus with Tools from Quantum Optimal Control," Quantum **6**, 824 (2022).
[14] Robert Friedman, "Real representations," `https://www.math.columbia.edu/~rf/realreps.pdf`.
[15] El Amine Cherrat, Snehal Raj, Iordanis Kerenidis, Abhishek Shekhar, Ben Wood, Jon Dee, Shouvanik Chakrabarti, Richard Chen, Dylan Herman, Shaohan Hu, Pierre Minssen, Ruslan Shaydulin, Yue Sun, Romina Yalovetzky, and Marco Pistoia, "Quantum Deep Hedging," Quantum **7**, 1191 (2023).
[16] Jeongwan Haah, Yunchao Liu, and Xinyu Tan, "Efficient approximate unitary designs from random pauli rotations," arXiv preprint arXiv:2402.05239 (2024).
[17] Aram W. Harrow and Richard A. Low, "Random quantum circuits are approximate 2-designs," Communications in Mathematical Physics **291**, 257–302 (2009).
[18] Fernando G. S. L. Brandão, Aram W. Harrow, and Michał Horodecki, "Local random quantum circuits are approximate polynomial-designs," Communications in Mathematical Physics **346**, 397–434 (2016).
[19] Jean Bourgain and Alex Gamburd, "A spectral gap theorem in $su(d)$," (2011), arXiv:1108.6264 [math.GR].
[20] Yves Benoist and Nicolas de Saxcé, "A spectral gap theorem in simple lie groups," Inventiones mathematicae **205**, 337–361 (2016).

Disclaimer

This paper was prepared for informational purposes by the Global Technology Applied Research center of JPMorgan Chase & Co. This paper is not a product of the Research Department of JPMorgan Chase & Co. or its affiliates. Neither JPMorgan Chase & Co. nor any of its affiliates makes any explicit or implied representation or warranty and none of them accept any liability in connection with this paper, including, without limitation, with respect to the completeness, accuracy, or reliability of the information contained herein and the potential legal, compliance, tax, or accounting effects thereof. This document is not intended as investment research or investment advice, or as a recommendation, offer, or solicitation for the purchase or sale of any security, financial instrument, financial product or service, or to be used in any way for evaluating the merits of participating in any transaction.
